# Public institutions for cleantech commercialization in the United States

**DOI:** 10.1038/s41467-026-76484-6

**Published:** 2026-08-13

**Authors:** Em Finkelstine, Laura Diaz Anadon, Jonas Meckling

**Affiliations:** 1 Department of Environmental Science, Policy, and Management, University of California, Berkeley, CA USA; 2 Centre for Environment, Energy and Natural Resource Governance, Department of Land Economy, University of Cambridge, Cambridge, UK; 3 Harvard Business School, Harvard University, Boston, MA USA

**Keywords:** Carbon and energy, Technology, Government, Climate-change mitigation

## Abstract

The United States has been building public institutions to promote cleantech commercialization since the 1970s oil crisis. The ascent of new industrial policy has accelerated this process substantially since 2020, resulting in a doubling of the total number of institutions by 2024. Introducing a dataset of 122 institutions, we show how institutions perform five functions: goal setting, coordination, derisking, imposing discipline, and external engagement. Further, we demonstrate how post-2020 institutions extend and diverge from historical trends, highlighting the recent buildout of planning capacity, the widespread use of conditionalities, and the pivot toward engagement with local and non-firm actors. We conclude with an analysis of how the second Trump administration is transforming the cleantech commercialization system through retrenching, redesigning, repurposing, and maintaining institutions. We offer a bottom-up, institution-level examination of the emergence and evolution of state capacity for green industrial policy in the United States, its functions, and current developments.

## Abstract (web summary)

This study maps U.S. public institutions for cleantech commercialization, showing how their functions have evolved during the recent turn toward green industrial policy.

## Introduction

Governments have increasingly relied on industrial policy to create and scale markets for clean technologies since the turn of the century. This has led to a wave of public institution-building for cleantech commercialization, complementing the historical focus on research and development (R&D) agencies^1^. This institutional growth has been a steady trend since the early 2000s and has accelerated since 2020^2^. The US Inflation Reduction Act and the EU’s Clean Industrial Plan are landmark policies reflecting this broader trend^3^.

Much research focuses on R&D policies to accelerate innovation in cleantech^4,5^, including questions about the design of R&D agencies such as the US Energy Frontier Research Centers^6–8^. Yet relatively little is known about the design of public institutions aimed at accelerating cleantech commercialization. At the same time, research on earlier periods of industrial policy in both advanced industrialized economies and late industrializers emphasizes that successful industrial policy hinges on the right institutions^9–17^. This knowledge gap—combined with the importance of commercializing cleantech to mitigate climate change^18^—calls for improving our systematic understanding of the design of institutions for green industrial policy.

Here, we examine public institutions for cleantech commercialization in the United States. The US has historically been the biggest funder of energy research, development and deployment (RD&D) in absolute terms^1^, but it has also increasingly created new institutions for cleantech commercialization, thus expanding public support beyond the early invention phase. We define commercialization institutions as programs or organizational units that manage or shape commercialization activities involving technologies at technology readiness levels (TRLs) 4 –10. This range corresponds to the demonstration and deployment stages of the innovation process, after research and development and before predictable growth. We introduce a dataset on the design of 122 US public institutions that aim to support and accelerate cleantech commercialization as one of their main goals. Our dataset includes institutions that were created between 1975 and 2024. The data reflect a cross-sectional view of the US institutions active in 2024, while we include comparisons of pre- and post-2020 institutions based on their start year (see Methods).

We code each institution along five major functions critical for successful technology commercialization: goal setting—providing strategic goals for government programs, coordination—eliminating obstacles to deployment via complementary technologies, infrastructure, policies, and inputs, derisking—providing funds to reduce market and technology risks, discipline—attaching conditionalities to funding, and external engagement—facilitating learning and resource-sharing through interaction with external actors (see Table 1 for definitions). These functions are derived deductively from the literature on industrial policy (see Table 2 in Methods), while several sub-functions were refined inductively during the coding process as recurrent features of institutional design. Within each major function, we include a series of sub-variables detailing an institution’s specific approach. Additionally, we record general information such as start year, sectoral focus, and mission statement. In total, each institution is coded along 129 dimensions (Supplementary Table 1), resulting in a total of 15,738 observations.

**Table 1:** Functions of public institutions for cleantech commercialization

Function	Definition	Examples
Goal setting	Developing strategy to support cleantech commercialization and/or establishing targets that are specific, measurable, and time-bound.	Energy Earthshots Initiative; Energy Storage Grand Challenge
Coordination	Eliminating bottlenecks that could impede scale-up and deployment.	Commercial Aviation and Alternative Fuels Initiative; Grid Deployment Office
Derisking	Contributing financial or in-kind support to decrease technology and market risk.	Loan Programs Office; tax credits
Discipline	Attaching conditionalities to financial or in-kind support.	Grant evaluation criteria; bonuses on IRA-era tax credits for meeting prevailing wage and apprenticeship requirements
External engagement	Involving outside actors in institutional processes to facilitate learning and contribute to program delivery.	Foundation for Energy Security and Innovation; Clean Cities and Communities Initiative

Table 2 in Methods provides key literature sources for each function.

We examine these institutions’ growth, governance, and scope before analyzing trends in goal setting, coordination, derisking, discipline, and external engagement. We then assess how the second Trump administration is transforming the cleantech commercialization system through retrenching, redesigning, repurposing, and maintaining institutions, and conclude with a discussion of broader trends and implications for future research.

## Results

### Growth, governance, and scope

**Fig. 1:**
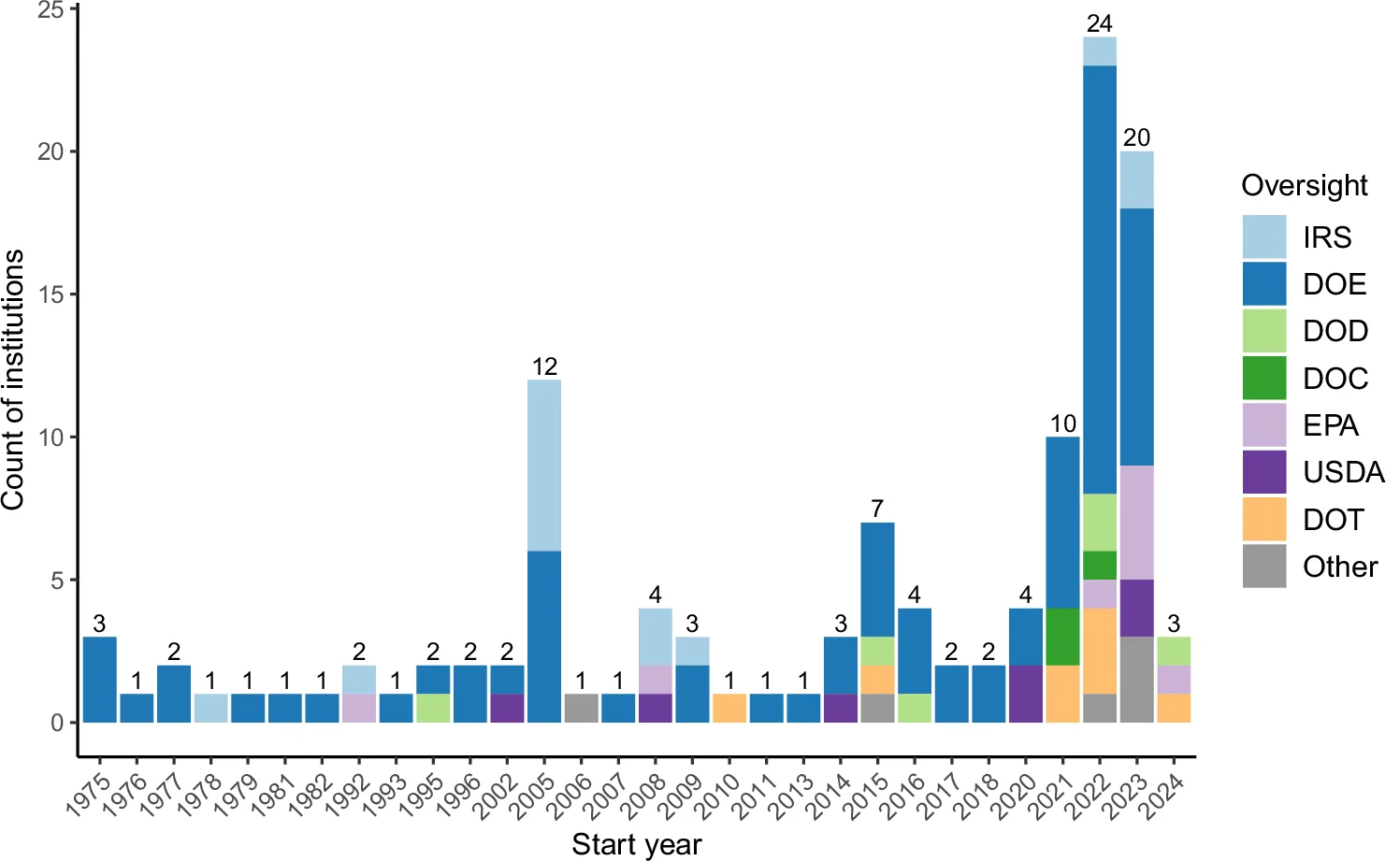
Count of US public institutions for cleantech commercialization by institution start year and oversight department. Light blue indicates IRS: Internal Revenue Service; dark blue indicates DOE: Department of Energy; light green indicates DOD: Department of Defense; dark green indicates DOC: Department of Commerce; light purple indicates EPA: Environmental Protection Agency; dark purple indicates USDA: United States Department of Agriculture; yellow indicates DOT: Department of Transportation. Gray indicates “Other,” which includes: Department of Housing and Urban Development, Department of the Interior, General Services Administration, National Science Foundation, and public-private partnerships with predominantly private sector management. Dataset includes 122 institutions. In this figure, oversight departments are mutually exclusive—institutions with multiple oversight departments have been assigned a priority department in the following order: IRS, DOE, DOD, DOC, EPA, NSF, USDA, DOT, Other. See Supplementary Fig. 1 for a breakdown of derisking funds by institution start year and oversight department. Source data are provided as a Source Data file.

We find major growth in US public institutions for cleantech commercialization since 2020, driven by the Bipartisan Infrastructure Law of 2021 and the Inflation Reduction Act of 2022. Out of 122 institutions, half were founded between 1974 and 2019, while the other half were founded in the five-year period between 2020 and 2024 (Fig. 1).

Over half (58.2%) of the total set of cleantech commercialization institutions are under the authority of the Department of Energy (DOE), followed by the Internal Revenue Service (11.5%), Department of Transportation (8.2%), US Department of Agriculture (6.6%), and Environmental Protection Agency (6.6%), with other institutions scattered across the Department of Commerce, National Science Foundation, Department of Defense (DOD), and Department of Housing and Urban Development (Supplementary Fig. 2). While the DOE remains dominant across time, we see a greater diversification in oversight departments beginning in the mid-2000s.

The primary policy missions of these institutions—according to their mission statements—are to address climate change (63.9%) and to increase US competitiveness and growth (57.4%). Two-thirds of institutions have more than one policy mission, and contributing to energy security and social equity are also important to over one-third of institutions. The post-2020 wave shows an increased concern with social equity (45.9% post-2020 vs. 24.6% pre-2020), competitiveness and growth (68.9%% post-2020 vs. 45.9% pre-2020), and climate change (68.9% post-2020 vs. 59% pre-2020) (Supplementary Fig. 3).

**Fig. 2:**
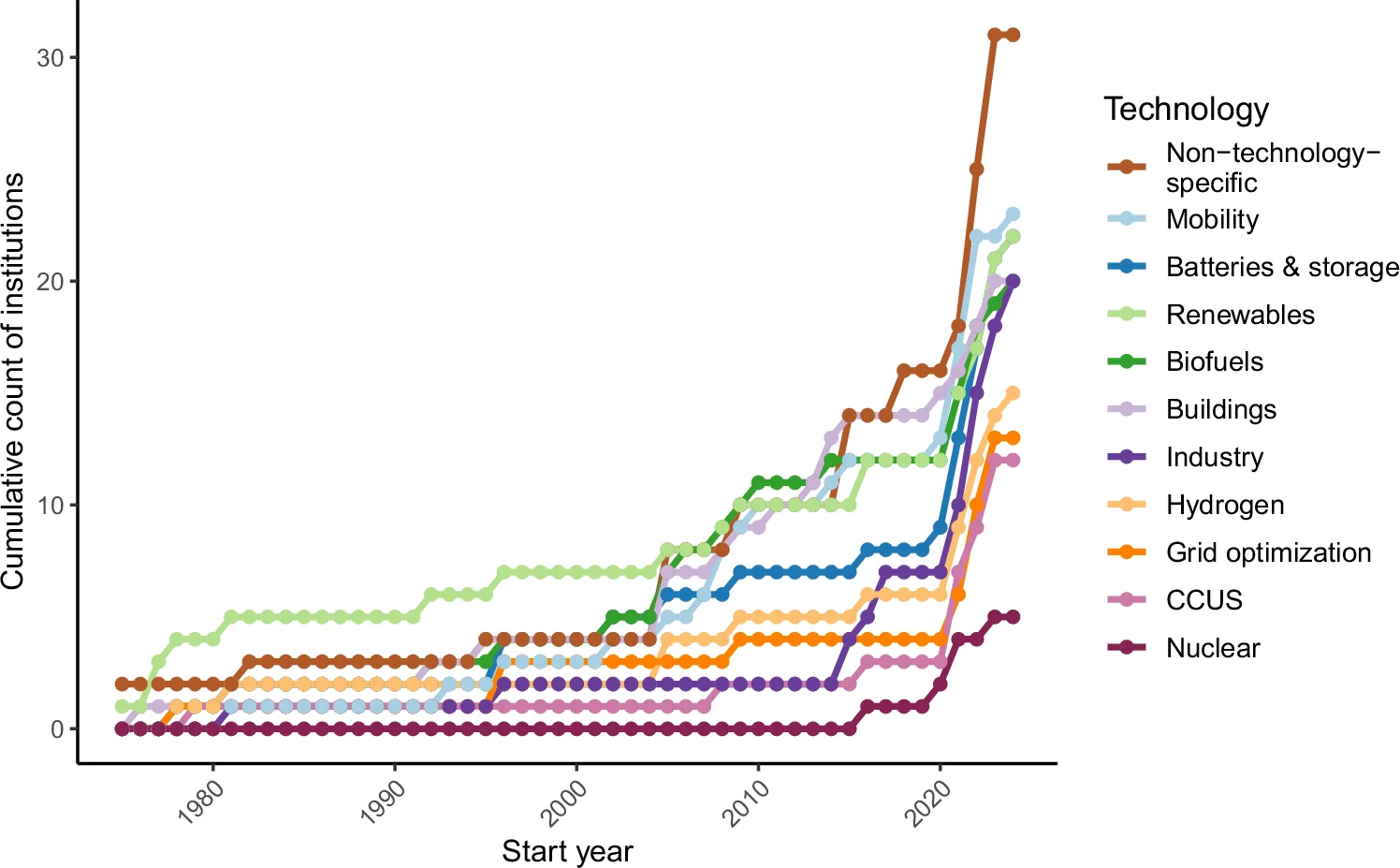
Cumulative count of US institutions’ technology focus areas, ordered by institution start year (1974–2024). Brown denotes non-technology-specific institutions, defined as institutions with broad technology coverage and/or objectives that span technology categories, such as building energy innovation ecosystems or developing clean energy manufacturing capacity. Light blue captures mobility institutions, indicating those working on technologies necessary for low-carbon or zero-carbon transport, excluding biofuels and batteries. Dark blue represents batteries and energy storage. Light green covers renewable energy, including solar, wind, ocean, geothermal, and hydroelectricity. Dark green covers biofuels, including liquid biofuels, solid biofuels, and biogases. Light purple refers to building technologies; dark purple to industrial process decarbonization; yellow to hydrogen and fuel cells; orange to grid optimization, including renewables integration and smart grids; and light magenta to Carbon Capture, Utilization, and Storage (CCUS). Dark magenta refers to nuclear technologies, limited to generation IV reactors and small modular reactors. Technology focus areas are not mutually exclusive. The dataset includes 122 institutions. See Supplementary Fig. 4 for cumulative derisking funds associated with technology focus areas, ordered by institution start year. Source data are provided as a Source Data file.

A quarter of institutions are non-technology-specific, meaning they focus on broad cross-sectoral interventions rather than specific technologies. These institutions provide general support for clean energy technology commercialization, or they focus on a specific theme such as building up the manufacturing base for clean energy technologies, supporting states and communities in deployment efforts, or providing support and mentorship to entrepreneurs. Three-quarters of institutions focus on specific sectors or technologies, most commonly: mobility (18.9%), batteries and energy storage (18%), renewables (18%), biofuels (16.4%), buildings (16.4%), and industry (16.4%) (Fig. 2). Mobility refers to the vehicles and enabling infrastructure required to facilitate low-carbon transportation, which we code separately from biofuels and batteries. Overall, mobility and mobility-adjacent institutions have a relatively high presence in our sample, reflecting this as a policy priority. In the post-2020 wave of institutions, we see a shift away from buildings (9.8% post-2020 vs. 23% pre-2020) and biofuels (13.1% post-2020 vs. 19.7% pre-2020) and a shift toward carbon capture, utilization and storage (CCUS) (14.8% post-2020 vs. 4.9% pre-2020), and batteries and energy storage (23% post-2020 vs. 13.1% pre-2020) (Supplementary Fig. 5). This reflects a changing of the guard as more mature technologies begin to see less focus, while emerging technologies take center stage.

### Goal setting and coordination

Goal setting and coordination provide direction and can help identify and eliminate obstacles for cleantech commercialization^19^. Goal setting is part of policy formulation and coordination is part of policy implementation. We define goal setting as the act of establishing targets that are specific, measurable, and time-bound. Targets may pertain to emissions reductions, sustainability criteria such as energy efficiency or recyclability, cost reductions, deployment benchmarks, or supply chain development. Institutions may also engage in goal setting by developing strategies for executing a project or commercializing a technology. Goal setting can serve as a signal to guide and align government and firm investments in clean energy technologies, and it can be used to orient multiple institutions across government around shared missions and strategic priorities^20–23^. For example, the Energy Earthshots Initiative sets time-bound cost targets for key clean energy technologies, including the Hydrogen Shot’s goal of one-dollar-per-kilogram clean hydrogen within the decade.

We define coordination as developing complementary technologies, building enabling infrastructure, creating or updating supportive policies, and addressing shortages in labor and raw materials to enable the scaling of technology commercialization^24–26^. For example, the Commercial Aviation Alternative Fuels Initiative (CAAFI) is a coalition of industry and government actors. It performs policy coordination by developing jet fuel certification processes that enable the deployment of sustainable aviation fuels.

**Fig. 3:**
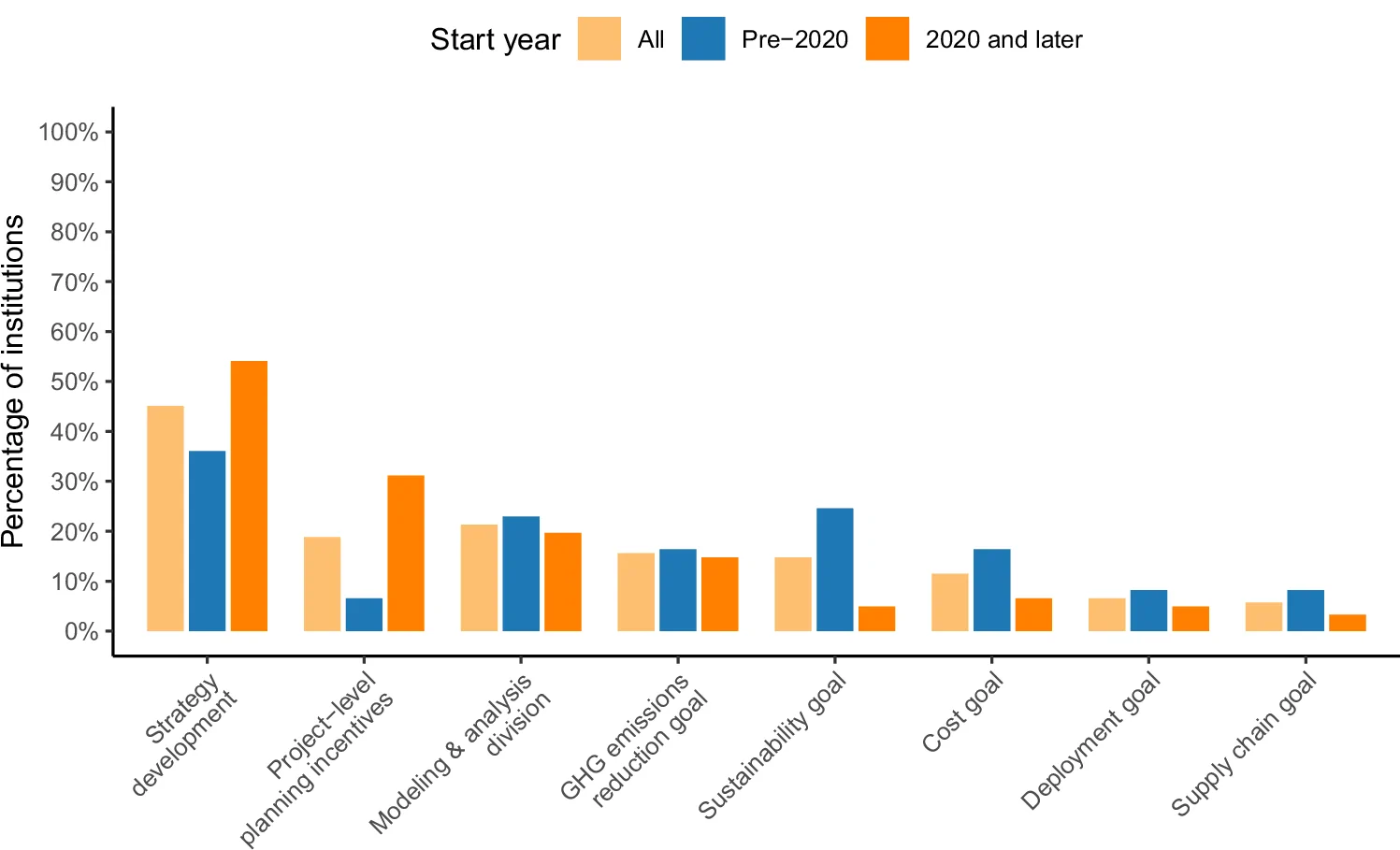
Percentage of institutions engaging in goal setting types, color coded by institution start year. Yellow bars report the total percentage of institutions performing each type of goal setting; blue bars report the percentage among institutions founded before 2020; and orange bars report the percentage among institutions founded in 2020 or later. The dataset includes 122 institutions: 61 founded before 2020 and 61 founded in 2020 or later. Goal setting types are not mutually exclusive. “Strategy development” denotes institutions that devise and publish strategies to guide cleantech commercialization, such as through a roadmap or multi-year program plan. “Project-level planning incentives” refers to requirements and support for detailed project planning as part of award structures. “Modeling & analysis division” indicates a dedicated team performing research to assess technology impacts or guide deployment. Other goal setting types include measurable, time-bound goals for GHG emissions reduction, sustainability (e.g., efficiency or recyclability), cost reduction, deployment benchmarks, or supply chain development. Source data are provided as a Source Data file.

Goal setting has the lowest prevalence of the five institutional functions. Just over half of institutions have published at least one specific, measurable, time-bound goal or engage in strategy development, project-level planning incentives, or modeling and analysis (Supplementary Fig. 6). Post-2020 institutions exhibit a higher degree of strategy development, meaning that they devise and publish strategies to support cleantech commercialization, such as through a roadmap or multi-year program plan (54.1% post-2020 vs. 36.1% pre-2020). More post-2020 institutions also include project-level planning incentives and support as part of award structures (31.1%) compared to pre-2020 institutions (6.6%) (Fig. 3). Many institutions with project-level planning incentives have a phased award structure, in which earlier phases are dedicated to project planning and later phases fund implementation. Others, especially Bipartisan Infrastructure Law programs that distribute formula funding to states for cleantech deployment, such as the Carbon Reduction Program and the National Electric Vehicle Infrastructure Program, require the submission of annual plans before funds are distributed. In both cases, institutions offer technical support to help funding recipients meet these requirements. The higher prevalence of this design feature in the post-2020 wave shows increased efforts to build planning capacity among non-federal actors, including firms, states, and communities.

**Fig. 4:**
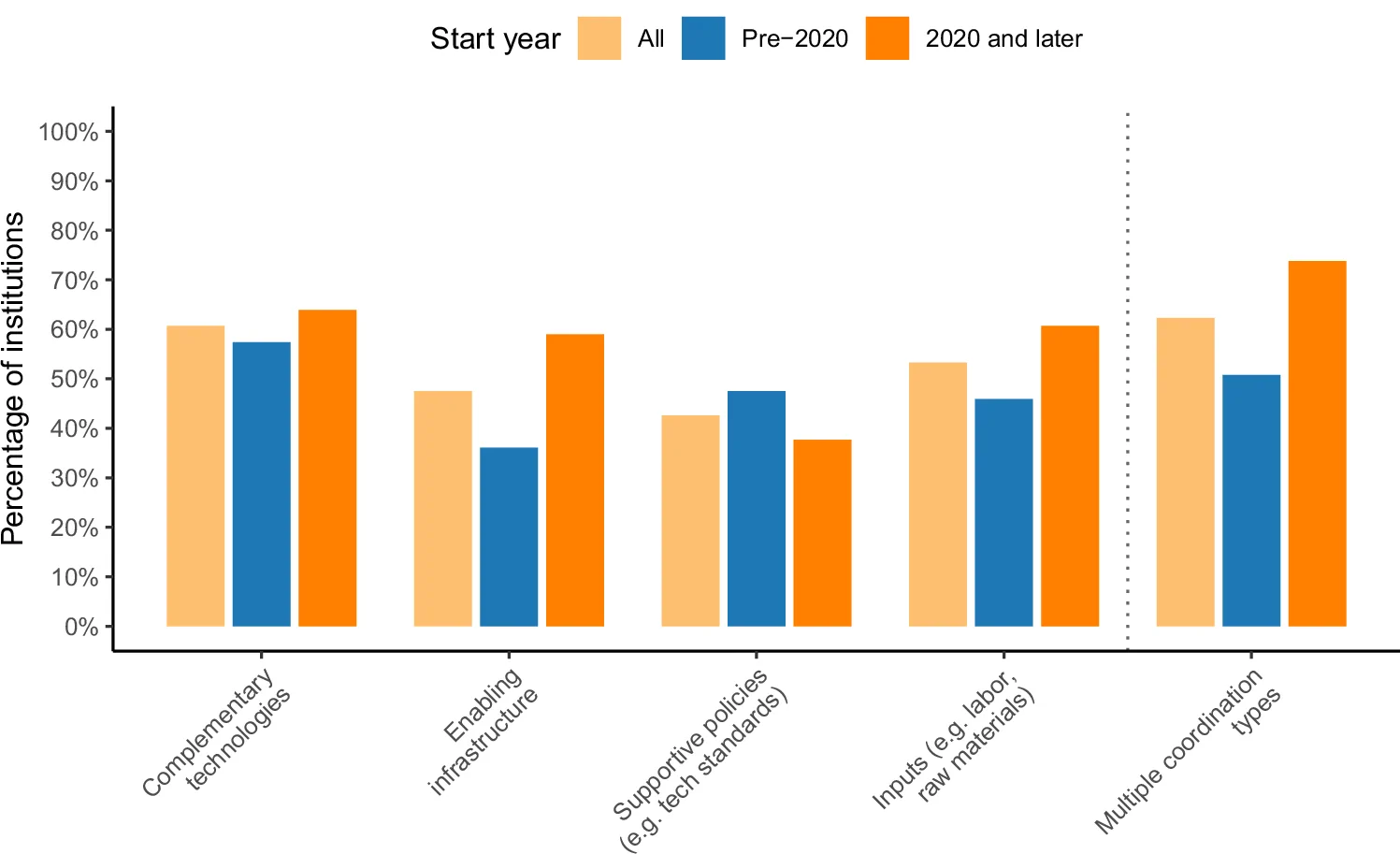
Percentage of institutions engaging in coordination types, color coded by institution start year. Yellow bars report the total percentage of institutions performing each type of coordination; blue bars report the percentage among institutions founded before 2020; and orange bars report the percentage among institutions founded in 2020 or later. The dataset includes 122 institutions: 61 founded before 2020 and 61 founded in 2020 or later. Coordination types are not mutually exclusive. “Multiple coordination types” means that an institution is performing two or more coordination types. See Supplementary Fig. 7 for derisking funds flowing through institutions performing each coordination type, color coded by institution start year. Source data are provided as a Source Data file.

The large majority of institutions (86.1%) are performing a coordination function (Supplementary Fig. 6), with over half of all institutions (62.3%) engaging in more than one type of coordination. In the post-2020 wave, we see a greater focus on building enabling infrastructure (59% post-2020 vs. 36.1% pre-2020) and addressing shortages in labor and raw materials (60.7% post-2020 vs. 45.9% pre-2020) (Fig. 4). Additionally, 73.8% of post-2020 institutions perform more than one form of coordination, while this is only true for 50.8% of pre-2020 institutions.

### Derisking and discipline

Derisking and discipline are the carrots and sticks that provide momentum and direction to government support for commercialization. We define derisking as any institutional action that reduces risks related to technology performance, costs, or market demand. Institutions can derisk with a primary focus on the supply side through technology-push measures that support RD&D or manufacturing scale-up, or on the demand side through demand-pull measures that increase demand for cleantech, including consumer tax incentives, government procurement, or deployment-oriented grants and loans^5,27,28^. Derisking can usher new technologies through the “valley of death,” i.e., the gap in funding between technology development and early deployment^29^. It can also improve the bankability of large-scale projects and mobilize private investment^30–32^. However, public institutions may risk the misuse of government money unless they pair derisking with discipline, also known as conditionalities: the strings attached to government funding and support. For example, institutions may require projects to meet technical standards or cost targets, or they may favor projects with higher emissions-reduction potential. Institutions may disqualify or disadvantage projects that fail to meet conditionalities during the application process or later cease funding or claw back funds if projects do not meet agreed-upon benchmarks. Discipline paired with derisking can work to prevent economic rent-seeking, enable governments to steer the direction of innovation, and direct the social and economic spillovers of innovation—including climate and distributional impacts—toward the public good rather than private gain^9,33–36^. In the context of multi-dimensional problems such as climate change and social inequality, governments can also use conditionalities to “multi-solve,” though this often involves trade-offs^37^.

Nearly all institutions (91%) carry out derisking (Supplementary Fig. 6), and this always co-occurs with a disciplinary function. Disciplinary functions include environmental conditionalities that guard against pollution, habitat disruption, and resource overuse; emissions conditionalities that prioritize technologies with greater avoided emissions; economic conditionalities that favor projects with stronger commercial viability; and social equity conditionalities that mandate community engagement and encourage projects in low-income areas. Local content requirements place restrictions on how many components or how much labor can be sourced internationally. Checkpoints require projects to meet milestones in order to retain their funding, and direct involvement means that an institution maintains at least some decision-making authority in the projects it funds, usually through a cooperative agreement structure.

**Fig. 5:**
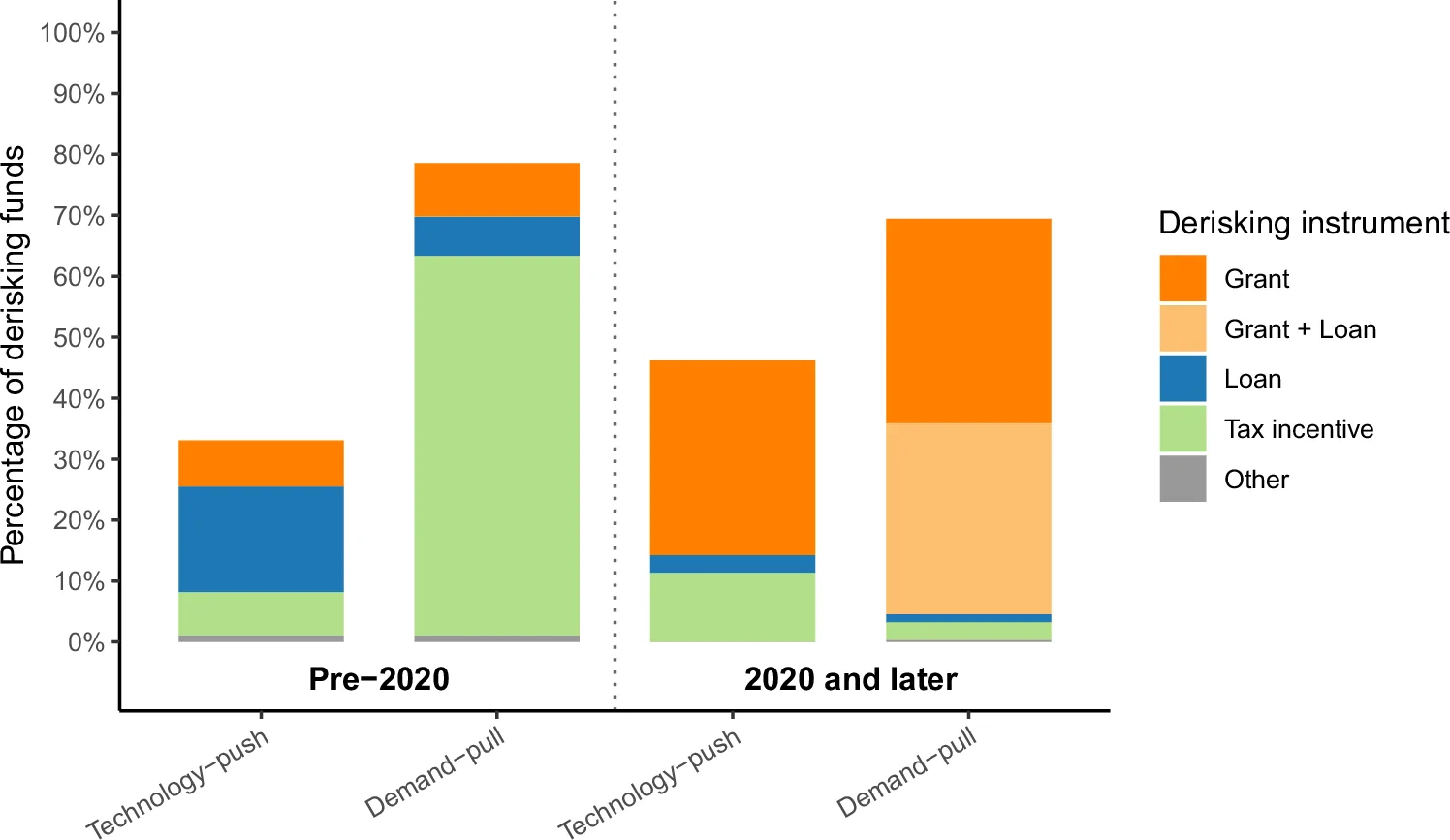
Percentage of derisking funds flowing through institutions performing each derisking type, separated by institution start year and color coded by derisking instrument. Percentages are calculated within each start year group, using the sum of institutions’ average annual derisking funds awarded from 2022 to 2024. Total derisking funds equal USD 91.07 billion across the full dataset of 122 institutions, USD 47.45 billion for institutions founded before 2020 (61 institutions), and USD 43.62 billion for institutions founded in 2020 or later (61 institutions). Technology-push derisking includes funding for RD&D, manufacturing, and scale-up, while demand-pull derisking increases demand or facilitates adoption. These derisking types are not mutually exclusive. For clarity, the figure is restricted to grants (orange), loans (blue), simultaneous use of grants and loans (yellow), and tax incentives (light green), as other derisking instruments (gray) account for only marginal funds. For a version of this figure with technology-push derisking disaggregated into RD&D and manufacturing scale-up, see Supplementary Fig. 8. Source data are provided as a Source Data file.

Institutions founded in 2020 or later show an increase in technology-push derisking (Fig. 5), while most derisking dollars continue to go toward demand-pull measures. This reflects both the incremental shift toward supply-side industrial policy and the cost intensity of demand-pull mechanisms. In the post-2020 wave of institutions, we also see derisking shift toward more grants and fewer tax incentives, both in terms of the percentage of institutions (Supplementary Fig. 9a) and the percentage of derisking funds (Supplementary Fig. 10). However, we note that many tax credits created before 2020 were later renewed under the IRA.

**Fig. 6:**
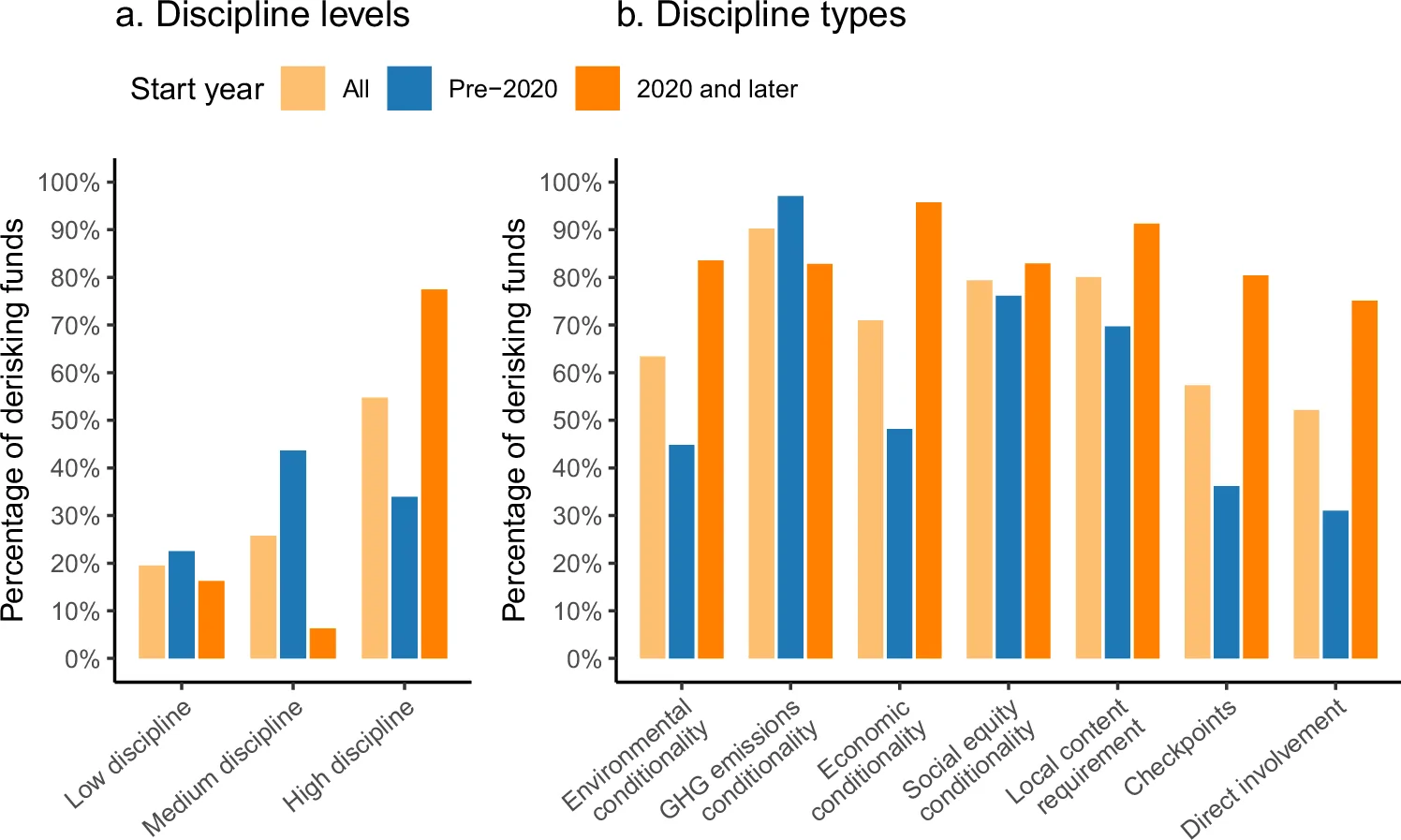
Discipline characteristics associated with derisking funds. **a** Percentage of derisking funds managed by institutions with different discipline levels, and **b** Percentage of derisking funds managed by institutions imposing different discipline types, color coded by institution start year. Derisking funds are measured as the sum of each institution’s average annual funds awarded over 2022–2024. Total derisking funds equal USD 91.07 billion across the full dataset of 122 institutions, USD 47.45 billion for institutions founded before 2020 (61 institutions), and USD 43.62 billion for institutions founded in 2020 or later (61 institutions). Yellow bars report the percentage of total derisking funds associated with institutions applying a given discipline level or type; blue bars report the percentage among institutions founded before 2020; and orange bars report the percentage among institutions founded in 2020 or later. While discipline levels are mutually exclusive, discipline types are not. Low discipline refers to institutions applying 1–2 conditionalities, medium discipline to 3–5 conditionalities, and high discipline to 6–7 conditionalities. Environmental conditionalities impose requirements on project impacts beyond greenhouse gas emissions; greenhouse gas (GHG) emissions conditionalities favor lower-emissions projects; economic conditionalities impose requirements related to commercial potential or commercial performance; and social equity conditionalities favor projects in low-income areas or areas more affected by the energy transition, or mandate labor standards or salary requirements. Checkpoints are points at which projects are evaluated so the institution can adjust course if necessary, including by clawing back funds. Direct involvement means that an institution retains some decision-making authority over its funded projects. Source data are provided as a Source Data file.

The post-2020 wave also exhibits a greater amount of derisking dollars subject to high discipline levels, meaning that 6–7 simultaneous conditionalities apply to these funds (Fig. 6a). This increase in disciplinary functions is driven by a higher percentage of derisking dollars tied to economic (95.7% post-2020 vs. 48.2% pre-2020) and environmental (83.6% post-2020 vs. 44.8% pre-2020) conditionalities. Local content requirements, checkpoints, and direct involvement likewise correspond to more than 20% higher derisking allocations among post-2020 institutions (Fig. 6b).

### External engagement

We define external engagement as the processes through which institutions involve outside actors in their decision-making, management, and program implementation. These outside actors may fall within the government (Congress, other public institutions, subnational governments), or they may be actors beyond the government (firms, investors, research institutions, nonprofits, communities, citizens). External engagement serves the core function of learning, given information asymmetries between the public and private sectors^38–40^. It can also engage the private sector to perform components of program delivery that the public sector lacks the knowledge or capacity to carry out directly^41^. Finally, external engagement can structure the relationships that underpin the developmental network state, with public institutions acting as trusted brokers to connect firms, investors, research institutions, and other actors with the complementary expertise and capabilities required to initiate and advance projects^12,14,42–45^.

**Fig. 7:**
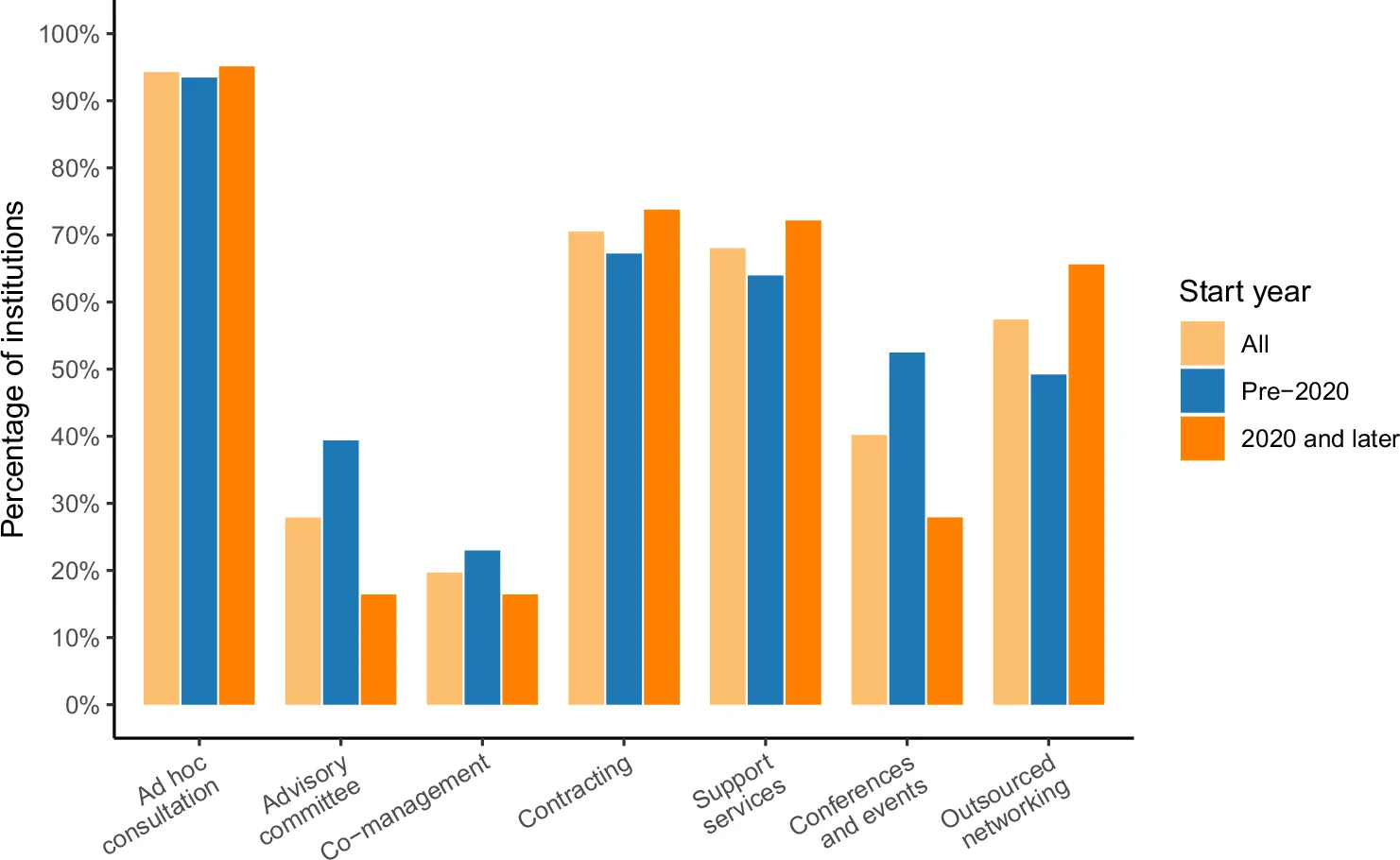
Percentage of institutions performing external engagement types, color coded by institution start year. Yellow bars report the total percentage of institutions with each characteristic; blue bars report the percentage among institutions founded before 2020; and orange bars report the percentage among institutions founded in 2020 or later. The dataset includes 122 institutions: 61 founded before 2020 and 61 founded in 2020 or later. External engagement types are not mutually exclusive. Ad hoc consultation refers to temporary, as-needed consultation, such as requests for information, listening sessions, or informal consultation. Advisory committees are long-term structures for soliciting stakeholder feedback. Co-management means that outside actors share in institutional governance or decision-making authority, for example through boards with private sector members. Contracting means that an institution uses partners to perform some aspects of program delivery. Support services are direct project assistance, such as expertise, mentorship, or access to government facilities, including national labs. Conferences and events convene stakeholders for mutual learning or exchange. Outsourced networking means that an institution encourages external actors to form networks, often by requiring funding applicants to apply as consortia or demonstrate buy-in from actors such as subnational governments or labor groups. Source data are provided as a Source Data file.

All institutions in our dataset carry out some form of external engagement (Supplementary Fig. 6), though this is partially driven by the prevalence of the lowest-effort method: ad-hoc consultation (94.3%), such as through requests for information or listening sessions (Fig. 7). However, 82% of institutions are involved in other forms of external engagement, including involving external actors in advisory committees, co-management (e.g., boards of directors), program delivery (e.g., contracting), or providing technical assistance to awardees. Greater breadth and depth of engagement are associated with institutions that engage in strategy development and institutions that perform policy coordination.

**Fig. 8:**
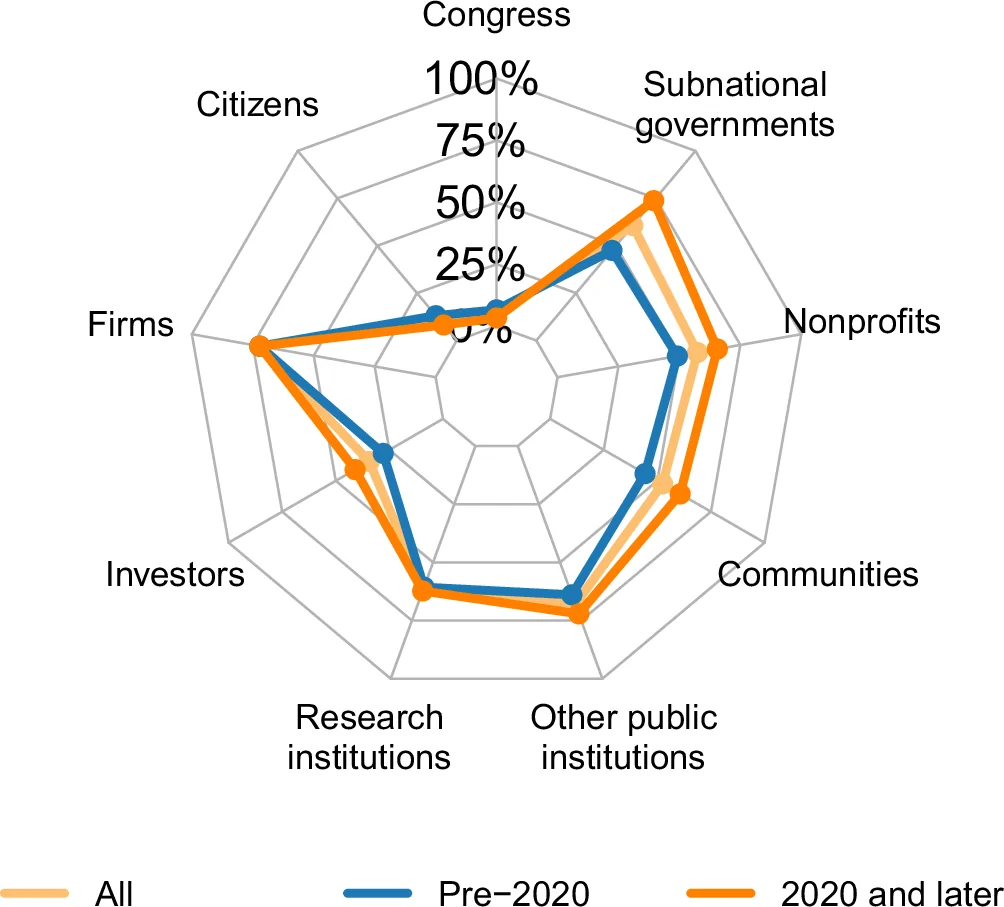
Percentage of institutions interacting with external engagement target groups, color coded by institution start year. Yellow lines report the total percentage of institutions engaging with each group; blue lines report the percentage among institutions founded before 2020; and orange lines report the percentage among institutions founded in 2020 or later. The dataset includes 122 institutions: 61 founded before 2020 and 61 founded in 2020 or later. External engagement target groups are not mutually exclusive. Source data are provided as a Source Data file.

The post-2020 wave of institutions shows greater involvement of almost every external actor group in our dataset. Subnational governments (73.8% post-2020 vs. 47.5% pre-2020), non-profits (65.6% post-2020 vs. 49.2% pre-2020) and communities (60.7% post-2020 vs. 44.3% pre-2020) have seen the biggest increase (Fig. 8). This coincides with an increase in outsourced networking in the post-2020 wave (65.6% post-2020 vs. 49.2% pre-2020), meaning that an institution incentivizes interactions between stakeholders (usually as a funding requirement) rather than working to build the network itself (Fig. 7). Pre-2020 institutions appear mostly focused on engaging the private sector. Post-2020 institutions emphasize outsourced networking that requires projects to interact directly with local governments, communities, and workers via statements of support, town hall meetings, or community benefits plans. In other words, private sector engagement is more commonly seen at the federal institution level, while local engagement is often negotiated at the project level.

### Institutional change under the second Trump administration

Since January 2025, the second Trump administration has taken steps to transform US public institutions for cleantech commercialization. We find that the administration is using four strategies of institutional change: (1) retrenching institutional capacity through funding and staffing cuts^46^, (2) redesigning institutions to eliminate design features inconsistent with the administration’s priorities^47^, (3) repurposing institutions to support favored energy technologies^48^, and (4) maintaining cleantech institutions^49,50^. Here, we illustrate each strategy, while evidence in Supplementary Table 2 demonstrates that these four strategies represent broader patterns of institutional change across the US green industrial state.

First, the second Trump administration is retrenching institutional capacity through funding and staffing cuts. As of October 2025, the administration has primarily cut IRA-authorized funding through the One Big Beautiful Bill Act (OBBBA), which attempts to rescind USD 93.4 billion from IRA climate and environment programs. Of these funds, one-third have been withdrawn, with the remainder subject to court rulings^51^. Many institutions have also lost large portions of their staff due to cuts orchestrated by the Department of Government Efficiency (DOGE). As of late April 2025, nearly 25% of DOE staff had resigned under DOGE’s deferred resignation program^46^.

Second, surviving institutions have also faced mandatory design changes, especially in the categories of discipline and external engagement. For instance, Trump’s January 2025 Executive Order on “Ending Radical and Wasteful Government DEI Programs and Preferencing” eliminates almost all social equity conditionalities. The order also targets external engagement with communities by eliminating the ability to require or enforce Community Benefits Plans for funded projects^52^.

Third, the second Trump administration is also attempting to repurpose some institutions such as the Office of Clean Energy Demonstrations (OCED) toward fossil fuels, nuclear, and critical minerals^53,54^. Though the office has been weakened through a USD 0 budget request for FY2026 and reorganization under the Office of Critical Minerals and Energy Innovation (CMEI), it continues to operate as a vehicle for the administration’s preferred energy investments. The DOE has canceled 61 OCED awards totaling over USD 12 billion, but the institution’s advanced nuclear and critical minerals projects remain untouched. Furthermore, the second Trump administration will use OCED to distribute USD 525 million in coal investments^48,55^.

Fourth, the second Trump administration is maintaining some cleantech institutions because they either advance its policy priorities or are insulated from substantial administrative influence. The administration has emphasized its support for select forms of renewable energy, namely biofuels, geothermal, and nuclear^56^. Accordingly, the OBBBA did not target tax credits in these areas, and even extended the biofuel credit^50^. DOE-based institutions in these technology areas are also slated for fewer funding cuts than their peers in other technology areas.

## Discussion

Here, we discuss the implications of our key findings on 1) growth, scope, and governance; 2) goal setting and coordination; 3) derisking and discipline; and 4) external engagement. We predominantly focus on trends in the dataset prior to the changes enacted under the second Trump administration.

First, the expansion of public institutions for cleantech commercialization in the US has been a steady process since the mid-2000s, accelerating substantially in 2020. We thus observe the emergence of state capacity for green industrial policy which aligns with the diffusion of green industrial policy measures^57^. Importantly, this new green state apparatus represents a “visible developmental state” as opposed to a “hidden developmental state” centered around the DOD that characterized earlier periods of industrial policy in the US^58,59^. The latter bolstered the US innovation system while largely avoiding the political opposition faced by more overt forms of industrial policy^16,41,58,60^. The 20-year buildout of the visible commercialization ecosystem for cleantech is now facing transformation through retrenching, redesigning, repurposing, and partially maintaining institutions. The unfolding institutional change reflects not only contraction but also transformation, with unclear outcomes.

Second, there are greater efforts to develop strategic planning capacity across public and private sectors. Post-2020 institutions engage to a greater extent in strategy development and focus increasingly on incentivizing and supporting the development of project-level planning capacity among funding recipients. In addition, coordination adopts a system-level approach to technology commercialization, with a focus on infrastructure and labor bottlenecks^61^. This shift toward system-level coordination could reflect the maturing of cleantech. The more widely new technologies are adopted, the more technology systems need to be reconfigured through coordination and infrastructure buildout as emphasized by the literature on innovation systems, technology transitions, and overcoming legacy sector lock-in^62–67^. In many cases, the private sector fails to solve these coordination problems, requiring the state to step in refs. ^26,68^.

Third, institutions for cleantech commercialization in the US have attached strings to public investments to a much higher degree (i.e., in all derisking programs) than portrayed by at least some analysts. Specifically, critics of US green industrial policy have argued that it represents a macro-financial regime that combines high derisking and low discipline—all carrots, no sticks^69^. Our findings suggest widespread use of conditionalities, which we refer to as the “disciplining” function, aligning with arguments made elsewhere^34^. Arguably, US conditionalities are quid pro quo, i.e., they apply to individual funding commitments, as opposed to the existence of broad regulatory requirements as in the European Union^70^. Firms also opt into many conditionalities to access additional funding.

Several factors have led to the greater prevalence of conditionalities in post-2020 institutions. The shift from tax credits to grants is one driver, as tax credits do not stipulate a National Environmental Policy Act review as a funding requirement, nor do they directly assess applicants’ commercialization potential. The rise of local content requirements likely reflects the Biden administration’s priority of boosting American manufacturing jobs^71^. Finally, the increase in checkpoints and direct involvement reveals stronger project oversight in the wave of new institutions to guard against misuse or waste of government money.

Fourth, the post-2020 institutions reflect a clear expansion in forms of external engagement from firms to non-firms and local actors. The growing efforts to promote engagement with local actors—including communities and nonprofits—are a necessity of place-based industrial policy, which features more prominently among post-2020 institutions. The pivot toward some non-firm actors could reflect a broadening of the goals the government pursues with industrial policy^72,73^. Green industrial policy is thus set in both a multi-goal and multi-constituency environment, requiring political bargains and tradeoffs across sets of interests. For example, in 2021, unions lobbied in favor of a conditionality on the IRA’s Clean Vehicle Credit that would require EVs to be manufactured at unionized plants in order to access USD 4500 of the USD 7500 total possible credit. This was met with strong resistance from European-headquartered auto firms without unionized workforces in the US. In the end, the unionization conditionality was eliminated in favor of a North American assembly requirement^74^.

Our findings have implications for future research. They raise questions about comparative lessons across jurisdictions, the origins of institutional design, and the effectiveness of institutions. The buildout of state capacity for green industrial policy is occurring across major economies. We need to understand how similar or different institutional designs are across countries and whether they reproduce national path dependencies in innovation systems. Cross-national comparisons with China and the EU are of particular interest, given the success of these economies in cleantech manufacturing and deployment. As for origin, future research needs to examine the politics of institution-building and institutional design, including the role of Congress, parent agencies, and external actors. The politics of a visible developmental state make these institutions more vulnerable, raising the question of how the political dynamics of repurposing existing institutions differ from those of building new ones. As regards the consequences of institutional design, a central question is which institutional designs are most effective at spurring technology commercialization by firms. This is a billion-dollar question as governments put taxpayer money to use in pursuit of economic and climate returns. Such analyses should focus not only on individual institutions but on ecosystems of institutions, given the possibility that complementary institutions may be most effective at supporting technology commercialization.

The rise of a visible developmental state also has implications for the politicization, durability and effectiveness of green industrial policy. A key feature of the post-War US innovation state has been its bipartisan support. This extended to cleantech RD&D funding, which both Democrats and Republicans backed in Congress—especially since the early 2000s^75^, including during the first Trump administration (2016–2020). Yet the post-2020 era suggests that public institutions for cleantech commercialization in the US may be increasingly subject to partisan swings as political polarization increases, heightening institutional volatility. While much of the innovation state was largely hidden from the public^58^, the industrial policy state is more visible and subject to public scrutiny, making it more vulnerable to partisan polarization and politicization.

Such partisan swings in institutional development are a problem for effective technology commercialization. To mitigate technology and market risks, public institutions need to be reliable long-term partners for firms. The current cancellations of planned investments by cleantech firms demonstrate the market impacts of institutional volatility, echoing the Reagan administration’s cutbacks of solar PV funding in the 1980s^76^. Stop-go cycles of institutional support create uncertainty that can delay market deployment substantially^77^. It also raises the specter of the potential loss of trust by market actors in public institutions, which could undermine the US government’s future ability to incentivize and steer technology commercialization.

## Methods

### Dataset construction and inclusion criteria

We identify institutions for the dataset by combing through legislation relevant to US green and cleantech industrial and commercialization policy, US government spending data, and the websites of government departments. For inclusion in the dataset, institutions must meet six criteria. First, institutions must dedicate at least 20% of their activities or a minimum of USD 50 million annually to cleantech commercialization. This threshold allows for the inclusion of larger institutions whose mandate extends beyond cleantech, but whose overall budgets are sufficiently large to make a substantial contribution to cleantech commercialization.

We adapt our cleantech categories from the IEA’s Clean Energy Technology Guide^78^. The technology areas include batteries and energy storage; CCUS; buildings; grid optimization; hydrogen and fuel cells; industrial decarbonization; mobility decarbonization; renewables, including geothermal energy, hydroelectricity, ocean energy, solar energy, and wind energy; biofuels, including liquid biofuels, solid biofuels, and biogases; and nuclear energy. We define grid optimization as technologies that reduce emissions from grid operations by improving efficiency, increasing flexibility, or enabling renewable energy integration. We code biofuels and batteries separately from mobility, and include only generation IV reactors and small modular reactors in the nuclear energy category. We code an institution as “non-technology-specific” if it supports at least 10 of the 14 technology groups used in the coding scheme or if it does not impose explicit limits on eligible technologies.

We exclude natural climate solutions, alternative proteins, incumbent technologies (TRL 11), fossil fuel-based heat and generation technologies, and institutions that exclusively focus on raw materials. Our definition of commercialization includes technologies between TRL 4 (validation in a laboratory environment) and 10 (integration at scale)^79,80^. Institutions active at lower or higher TRLs may still be included in the dataset as long as 20% or more of their activities, or USD 50 million or more of their budget, are focused on cleantech within this TRL range.

Second, we include only programs or organizational units that shape or manage activities. An institution shapes or manages activities if it has dedicated staff or has published a strategic plan, annual report, or website that details its mission and approach. We also include institutions without a designated staff or strategy when they manage over USD 100 million in funding, as this indicates that there are likely dedicated management procedures, even if these are not openly published.

Third, institutions must be public, meaning that they were established by the US government or by the US government in collaboration with private partners. An institution also qualifies as public if the US government provides at least 50% of its funding. We exclude institutions run by lower levels of government (i.e., state or city governments) without federal involvement.

Fourth, institutions must have been in existence as of 2024, though founding years range from 1975 to 2024 (Fig.1). Fifth, institutions must be relevant, which we distinguish by function. Institutions with a primary goal setting or coordination function are always coded, given that they meet the other inclusion criteria. Institutions with a primary external engagement function must have over 50 partners or network members. Institutions that primarily derisk through grants, other subsidies, and tax incentives must distribute over USD 10 million annually, while institutions that primarily derisk through loans, loan guarantees, or equity must distribute over USD 100 million annually.

We apply limited exceptions in three cases. First, institutions with disbursement levels below the threshold are included if they provide direct commercialization support, such as accelerators. Second, institutions that did not disburse funds during the 2022–2024 measurement period are included if their authorized budgets exceed the relevant threshold. For example, the Carbon Dioxide Transportation Infrastructure Finance and Innovation (CIFIA) program did not award funds during this period despite having a USD 2.1 billion budget, reflecting a delayed start-up phase. Finally, institutions that operate primarily through procurement or loan guarantees are included even when disbursement amounts for 2022–2024 cannot be consistently recorded, as such data are often not reported in comparable form.

Finally, we only code institutions for which there is sufficient publicly available information to allow for confident coding of their functions.

We code both parent institutions (for example, the Office of Energy Efficiency and Renewable Energy, EERE) and their sub-institutions (for example, the Solar Energy Technology Office within EERE) when both meet the thresholds for inclusion and parent institutions perform functions that go beyond their sub-institutions. When we code both a parent institution and its sub-institutions, we do not include the activities of the sub-institutions in our evaluation of the parent institution to avoid double-coding, except for when the parent institution plays a substantial role in helping the sub-institution carry out its functions. For example, derisking and discipline functions attributed to the sub-institution may also be coded in the parent institution if the management of derisking funds and the implementation of monitoring protocols are at least partially carried out by the parent. Supplementary Note 1 shows the institution selection criteria in list form, and Supplementary Note 2 shows the full list of selected institutions.

### Coding scheme

We develop a coding scheme to evaluate institutional design based on a review of the literature on technology commercialization and industrial policy in political science, sociology, innovation studies and economics. While we develop most variables based on their theoretical importance in the literature, we also expand the coding scheme to include variables that we inductively identify through the coding process. The coding scheme is built around the five major functions of goal setting, coordination, derisking, discipline, and external engagement. Sub-variables then give additional detail as to an institution’s approach to each applicable function. Most variables are binary. Coders mark 1 when an institution’s publicly available materials indicate the presence of a design feature. A value of 0 is assigned when the materials explicitly state that the institution does not perform the function or when no evidence of the function is found. Details on the coding process are provided in the next sub-section. Table 2 provides an overview of the functions and sub-variables along with their key references. Supplementary Table 1 shows the full coding scheme.

**Table 2:** Coding framework of institutional design functions

Function	Definition	Sub-types	Key references
Goal setting	Developing strategy to support cleantech commercialization and/or establishing targets that are specific, measurable, and time-bound.	• Strategy development: published strategy doc. since 2020 • Project-level planning incentives: technical support and requirements for planning integrated in award structure • Modeling & analysis division: dedicated team performing research to assess technology impacts or guide deployment • GHG emissions reduction goal • Sustainability goal (e.g., energy efficiency; recyclability) • Cost goal • Deployment goal • Supply chain goal (e.g., manufacturing capacity; reskilling)	Anadón^21^; Mazzucato^23^; Meckling and Nahm^20^; Hekkert et al.^19^
Coordination	Eliminating bottlenecks that could impede scale-up and deployment.	• Complementary technologies • Enabling infrastructure • Supportive policies (e.g., tech standards, permitting reform) • Inputs (e.g., labor, raw materials)	Markard and Hoffmann^24^; Markard, Geels, and Raven^25^; Rogge and Goedeking^81^
Derisking	Contributing financial or in-kind support to decrease technology and market risk.	Derisking Types • Tech-push: RD&D; tech-push: production; demand-pull • Technology risk, market risk Derisking instruments • Tax incentives • Grants (all) and cost-share grants; other subsidies • Loans (all) and partial loans; loan guarantees • Procurement • Equity	Nemet et al.^29^; Deleidi et al.^30^; Probst et al.^31^; Doblinger et al.^44^; Howell^82^
Discipline	Attaching conditionalities to funding or in-kind support.	Environmental conditionality (e.g., NEPA review; pollution restrictions besides GHGs) • GHG emissions conditionality • Economic conditionality (e.g., favoring projects with higher commercial potential) • Social equity conditionality (e.g., favoring low-income areas) • Local content requirement • Project checkpoints (e.g., go/no-go decision points) • Direct involvement (i.e., institution involved in project decisions)	Mazzucato and Rodrik^34^; Bulfone et al.^35^; Bulfone et al.^33^; Estevez^37^
External engagement	Interacting with outside actors in institutional processes to facilitate learning and improve program delivery.	Target group • Firms • Investors (e.g., private investors, banks, financial institutions) • Non-profits (e.g., trade unions, advocacy groups) • Research institutions (e.g., universities, think tanks, natl. labs) • Communities (i.e., groups in a location relevant to a project) • Citizens (i.e., households and individuals) • Congress • Subnational governments • Other public institutions Engagement type • Ad-hoc consultation (e.g., requests for information) • Advisory committee • Co-management (e.g., board of director members from outside the public sector; intermediaries managing funds) • Contracting or outsourcing • Support services (e.g., technical assistance; business services) • Conferences and events Special characteristics • Network building: directly facilitated or outsourced to recipients via funding requirements • Place-based program design (i.e., targeting and tailoring initiatives to local conditions)	Ó Riain^12^; Rodrik^38^; Fuchs^42^; Weiss^41^; Block and Keller^14^; Block et al.^43^; Hekkert et al.^19^

### Coding process

We carried out the coding process from January 2024 through December 2024 with a team of five coders and one supervisor. To ensure consistency, we developed a coding manual detailing the criteria for each variable and providing examples. The supervisor then guided coders through a training program during which all coders and the supervisor coded the same institution based on the coding manual, met to discuss their choices, and resolved any discrepancies. This process was repeated once more to ensure inter-coder reliability before coders began coding separate institutions. Throughout the data collection process, the supervisor reviewed all coding choices to ensure adherence to the coding manual. For each coding choice, coders were required to provide a source and a brief comment explaining their reasoning.

Coders reviewed each institution’s website and public materials to evaluate each variable, with most information coming from workplans, annual reports, program and project descriptions, press releases, IRS guidelines where applicable, and Notification of Funding Opportunity (NOFO) or Funding Opportunity Announcement (FOA) documents. When available, coders also consulted secondary sources from the academic literature with relevant information about the institution. We are therefore limited by the information institutions choose to publish, which constrains our dataset in two ways. First, our coding reflects institutions’ stated goals and activities in public materials rather than verified implementation. For example, it is unlikely to capture informal external engagement or disciplinary functions that may be more flexible in practice than in published guidelines. Second, we can only include institutions that publicly disclose their activities. As a result, we likely underestimate commercialization support within the DOD, where many activities are too opaque to be systematically coded. This limitation does not affect the central focus of the paper, which is to examine institutions for green industrial policy centered on the DOE, rather than to assess changes in the role of the DOD.

### Statistics and reproducibility

This study uses an original observational dataset of public institutions rather than an experimental design. No statistical method was used to predetermine sample size. The final sample reflects all institutions that met the inclusion criteria as detailed above. Institutions were excluded only when they fell outside the criteria. Because no experiments were conducted, randomization, allocation to experimental groups, and blinding were not applicable.

The analysis is descriptive and reports counts, percentages, and cross-period comparisons of institutional design features. Data was collected in Google Sheets. Analyses and visualizations were conducted in R 4.5.1 using dplyr, tidyr, ggplot2, waffle, fmsb, and ComplexUpset. Reproducibility was supported through a shared coding manual, coder training, source documentation for each coding decision, and supervisor review of all coding choices.

### Evaluating change over time

For parts of our analysis, we evaluate the percentage of institutions founded before 2020 versus the percentage of institutions founded in 2020 or later that exhibit a given design feature. We chose 2020 as the year to split our sample because it indicates a new wave of institutional expansion, in particular under the Biden administration (see Fig. 1). Conveniently, this also splits our sample exactly in half, with 61 institutions founded before 2020 and 61 institutions founded in 2020 or later.

Note that some institutions founded before 2020 adopted new design features after that date. For example, the LPO’s Title 17 Clean Energy Financing program, established in 2005, only adopted a Community Benefits Plan requirement under the Biden Administration, when this became a condition for distributing IRA funding through the DOE. In such cases, the design feature is attributed to the pre-2020 wave according to the institution’s start year, even though the design feature itself emerged after 2020. This results in an under-estimation of post-2020 trends. Thus, we provide conservative estimates of trends in the 2020–2024 wave of US green industrial policy relative to the preceding four decades of institutional design for cleantech commercialization.

### Reporting summary

Further information on research design is available in the Nature Portfolio Reporting Summary linked to this article.

## Supplementary information

Supplementary Information

Reporting Summary

Transparent Peer Review file

## Source data

Source Data

## Data Availability

The raw institutional design dataset is currently under restricted access due to additional ongoing analysis by the authors. Source data are provided with this paper.

## Code availability

The code used to generate Figs. 1–8 and Supplementary Figs. 1–10 is available upon request.
