# Supplementary material for: Public institutions for cleantech commercialization in the United States
Source: Nat Commun. 2026 Aug 13;17:9725. doi: 10.1038/s41467-026-76484-6 (PMC13562745; doi:10.1038/s41467-026-76484-6)
Supplement: Supplementary file 1 [file 41467_2026_76484_MOESM1_ESM.pdf]

Supplementary Information
for
Public institutions for cleantech commercialization in the United States

Em Finkelstine, Laura Diaz Anadon, Jonas Meckling
Correspondence to: meckling@berkeley.edu

Supplementary Fig. 1. Derisking funds flowing through institutions by institution start year and oversight department. Derisking funds are measured as the sum of each institution's average annual funds awarded over 2022–2024. Total derisking funds equal USD 91.07 billion across the full dataset of 122 institutions. Light blue indicates IRS: Internal Revenue Service; dark blue indicates DOE: Department of Energy; light green indicates DOD: Department of Defense; dark green indicates DOC: Department of Commerce; light purple indicates EPA: Environmental Protection Agency; dark purple indicates USDA: United States Department of Agriculture; yellow indicates DOT: Department of Transportation. Grey indicates "Other," which includes: Department of Housing and Urban Development, Department of the Interior, General Services Administration, National Science Foundation, and public-private partnerships with predominantly private sector management. In this figure, oversight departments are mutually exclusive—institutions with multiple oversight departments have been assigned a priority department in the following order: IRS, DOE, DOD, DOC, EPA, NSF, USDA, DOT, Other. Source data are provided as a Source Data file.

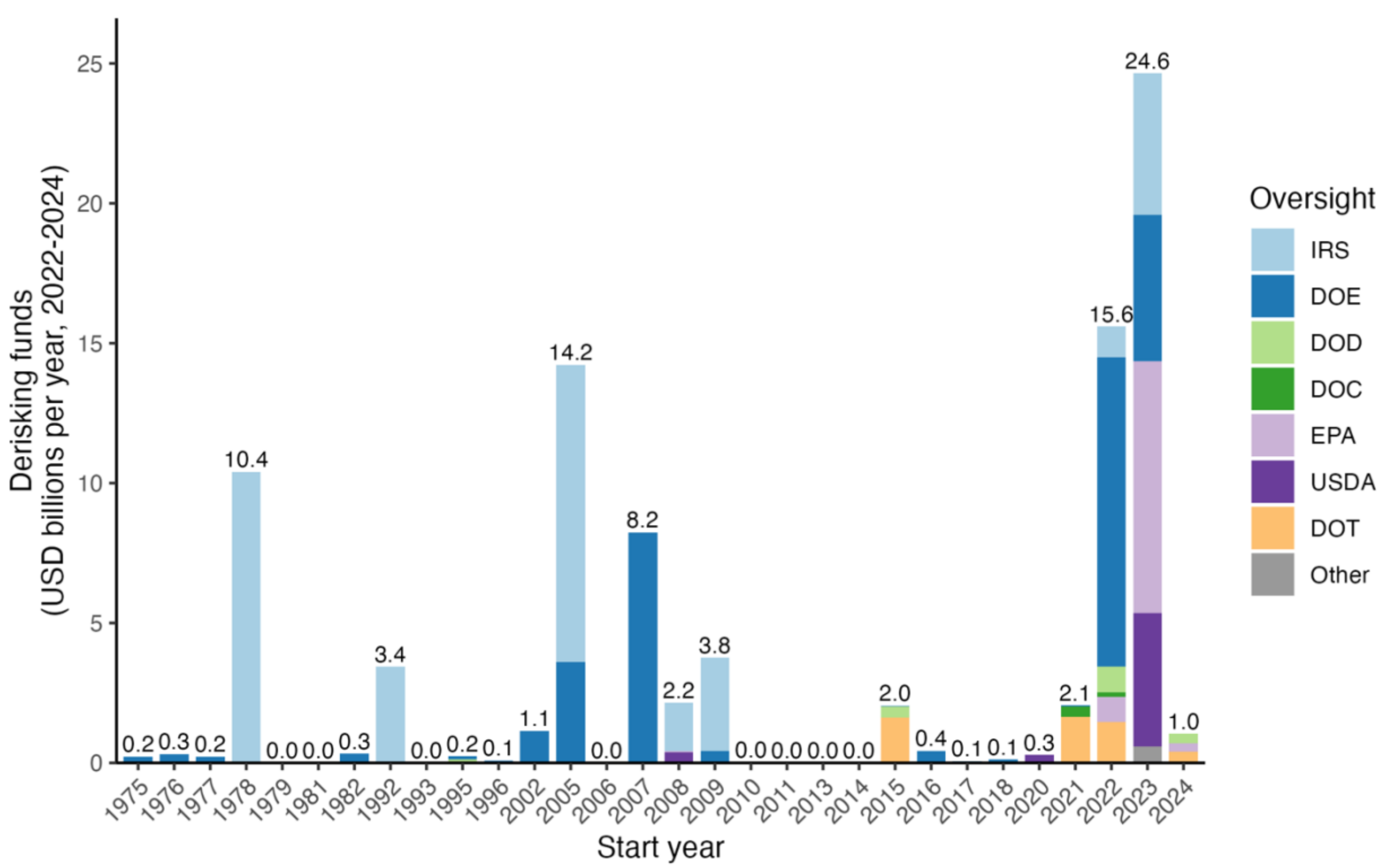

Supplementary Fig. 2. Percentage of institutions under each oversight department, color coded by institution start year. Yellow bars report the total percentage of institutions under each oversight department; blue bars report the percentage among institutions founded before 2020; and orange bars report the percentage among institutions founded in 2020 or later. The dataset includes 122 institutions: 61 founded before 2020 and 61 founded in 2020 or later. Oversight departments are not mutually exclusive, though most institutions fall under a single department. DOE: Department of Energy; IRS: Internal Revenue Service; DOT: Department of Transportation; EPA: Environmental Protection Agency; USDA: United States Department of Agriculture; DOD: Department of Defense; DOC: Department of Commerce; GSA: General Services Administration; DOI: Department of the Interior; NSF: National Science Foundation; HUD: Department of Housing and Urban Development; Independent: public-private partnerships with predominantly private sector management. Source data are provided as a Source Data file.

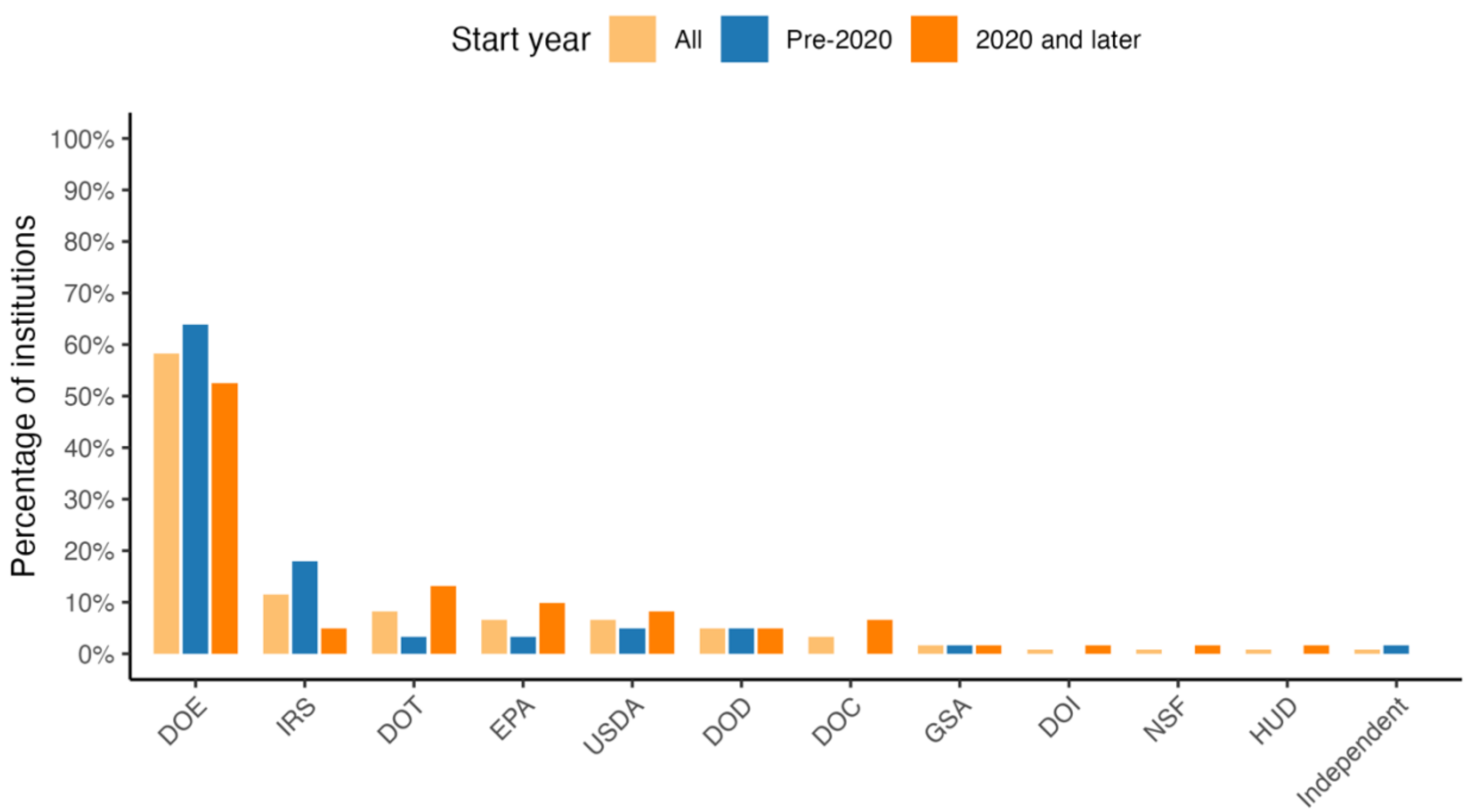

Supplementary Fig. 3. Percentage of institutions with each policy mission type, color coded by institution start year. Yellow bars report the total percentage of institutions with each policy mission; blue bars report the percentage among institutions founded before 2020; and orange bars report the percentage among institutions founded in 2020 or later. The dataset includes 122 institutions: 61 founded before 2020 and 61 founded in 2020 or later. Policy missions are not mutually exclusive. Source data are provided as a Source Data file.

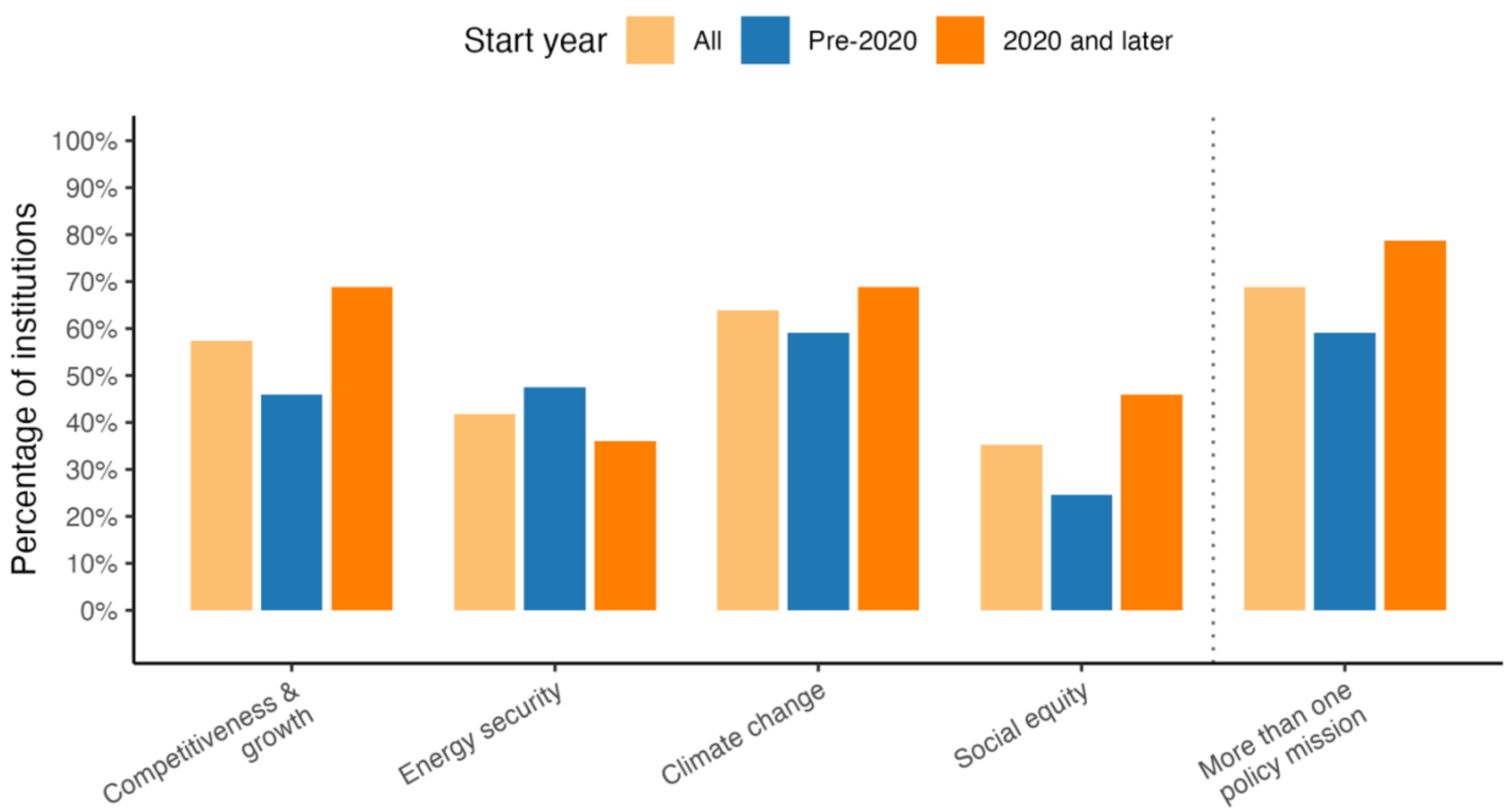

Supplementary Fig. 4. Cumulative derisking funds managed by institutions in technology focus areas, ordered by institution start year. Derisking funds are measured as the sum of each institution's average annual funds awarded over 2022–2024. Total derisking funds equal USD 91.07 billion across the full dataset of 122 institutions. Light green covers renewable energy, including solar, wind, ocean, geothermal, and hydroelectricity. Dark blue represents batteries and energy storage. Brown denotes non-technology-specific institutions, defined as institutions with broad technology coverage and/or objectives that span technology categories, such as building energy innovation ecosystems or developing clean energy manufacturing capacity. Yellow refers to hydrogen and fuel cells. Orange indicates grid optimization—technologies that reduce emissions from grid operations by improving efficiency, increasing flexibility, and enabling renewable energy integration. Light blue captures mobility institutions, indicating those working on technologies necessary for low-carbon or zero-carbon transport, excluding biofuels and batteries. Dark green covers biofuels, including liquid biofuels, solid biofuels, and biogases. Dark purple refers to industrial process decarbonization. Light magenta represents Carbon Capture, Utilization, and Storage (CCUS). Light purple covers building technologies. Dark magenta indicates nuclear technologies, limited to generation IV reactors and small modular reactors. Technology focus areas are not mutually exclusive. Source data are provided as a Source Data file.

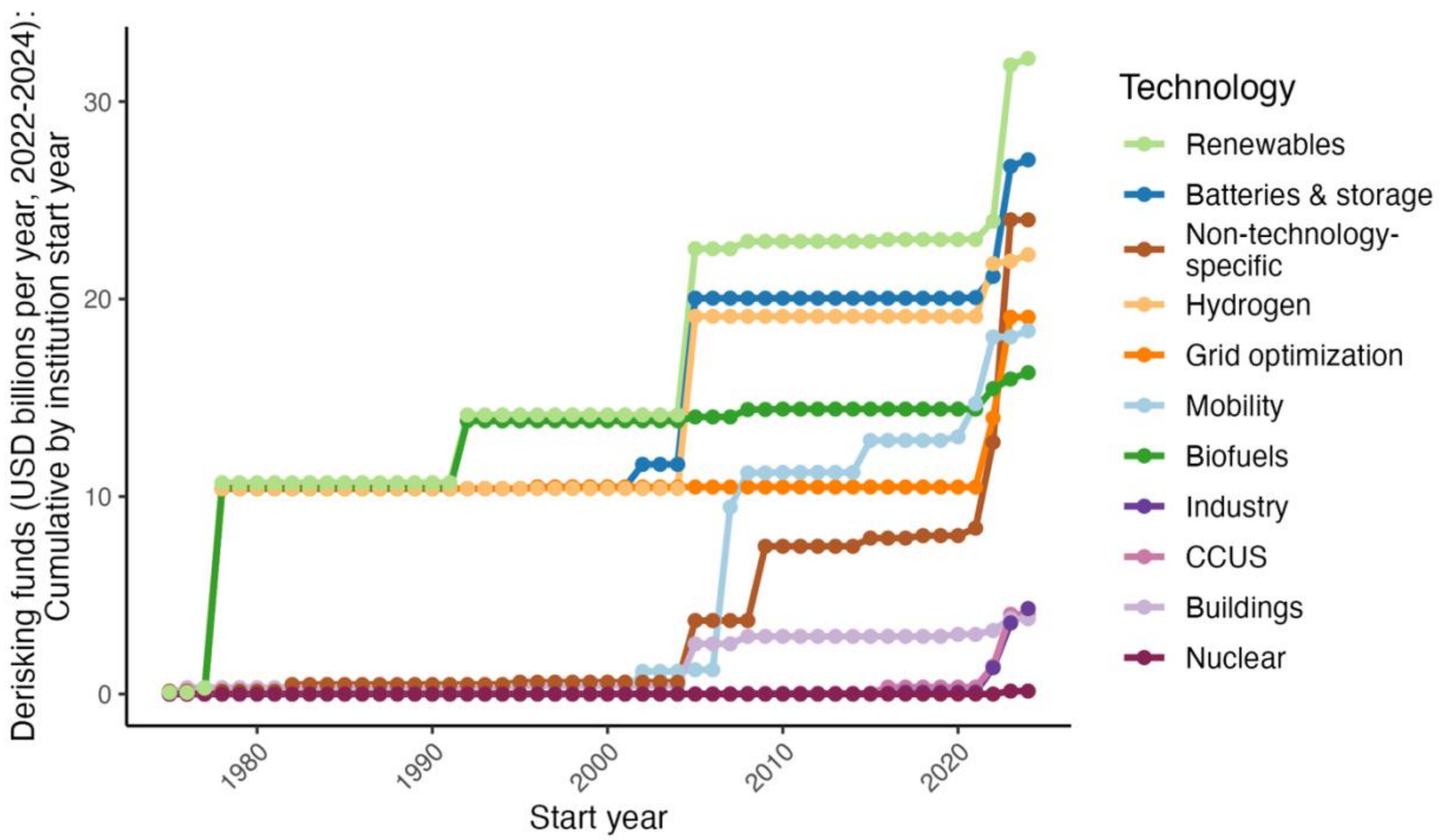

Supplementary Fig. 5. Percentage of institutions involved in technology focus areas, color coded by institution start year. Yellow bars report the total percentage of institutions working in each technology area; blue bars report the percentage among institutions founded before 2020; and orange bars report the percentage among institutions founded in 2020 or later. The dataset includes 122 institutions: 61 founded before 2020 and 61 founded in 2020 or later. Non-technology-specific institutions have broad technology coverage and/or objectives that span technology categories, such as building energy innovation ecosystems or developing clean energy manufacturing capacity. Mobility institutions cover technologies necessary for low-carbon or zero-carbon transport, excluding biofuels and batteries. Renewables include solar, wind, ocean, geothermal, and hydroelectricity. Biofuels include liquid biofuels, solid biofuels, and biogases. Grid optimization includes technologies that reduce emissions from grid operations by improving efficiency, increasing flexibility, and enabling renewable energy integration. Carbon Capture, Utilization, and Storage (CCUS) includes technologies that capture, use, transport, or store carbon dioxide. Nuclear technologies are limited to generation IV reactors and small modular reactors. Technology focus areas are not mutually exclusive. Source data are provided as a Source Data file.

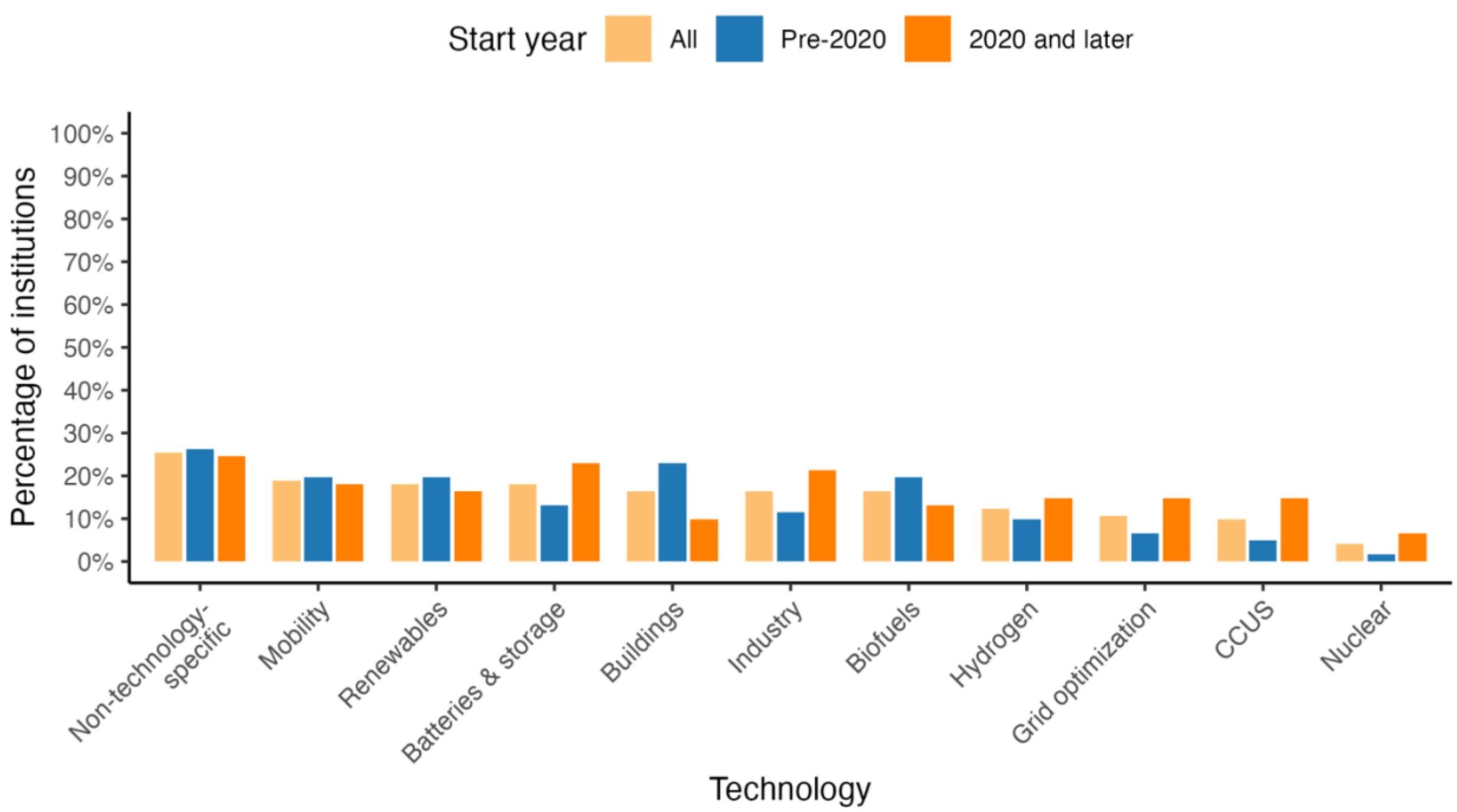

Supplementary Fig. 6. Percentage of institutions performing each of the five major functions, color coded by institution start year. Yellow bars report the total percentage of institutions performing each function; blue bars report the percentage among institutions founded before 2020; and orange bars report the percentage among institutions founded in 2020 or later. The dataset includes 122 institutions: 61 founded before 2020 and 61 founded in 2020 or later. Goal setting involves developing strategies to support cleantech commercialization and/or establishing targets that are specific, measurable, and time-bound. Coordination indicates efforts to eliminate bottlenecks that could impede scale-up and deployment. Derisking consists of financial or in-kind support designed to decrease technology or market risk. Discipline refers to conditionalities attached to financial or in-kind support. External engagement involves outside actors in institutional processes to facilitate learning and contribute to program delivery. Functions are not mutually exclusive. Source data are provided as a Source Data file.

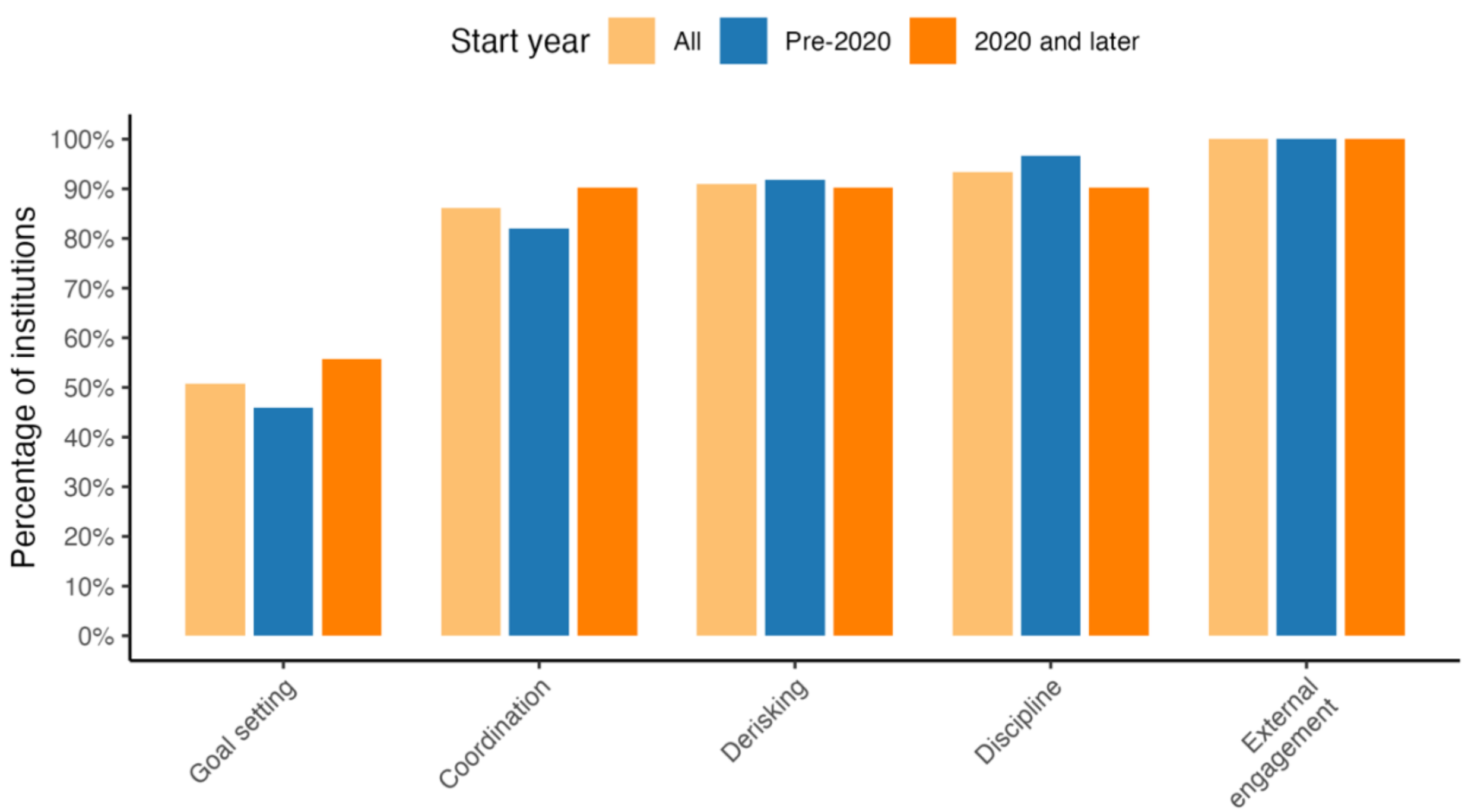

Supplementary Fig. 7. Derisking funds flowing through institutions performing each coordination type, color coded by institution start year. Yellow bars report the amount of total derisking funds flowing through institutions performing a given coordination type; blue bars report the amount among institutions founded before 2020; and orange bars report the amount among institutions founded in 2020 or later. Derisking funds are measured as the sum of each institution's average annual funds awarded over 2022–2024. Total derisking funds equal USD 91.07 billion across the full dataset of 122 institutions, USD 47.45 billion for institutions founded before 2020 (61 institutions), and USD 43.62 billion for institutions founded in 2020 or later (61 institutions). Coordination types are not mutually exclusive. "Multiple coordination types" means that an institution is performing two or more coordination types. Source data are provided as a Source Data file.

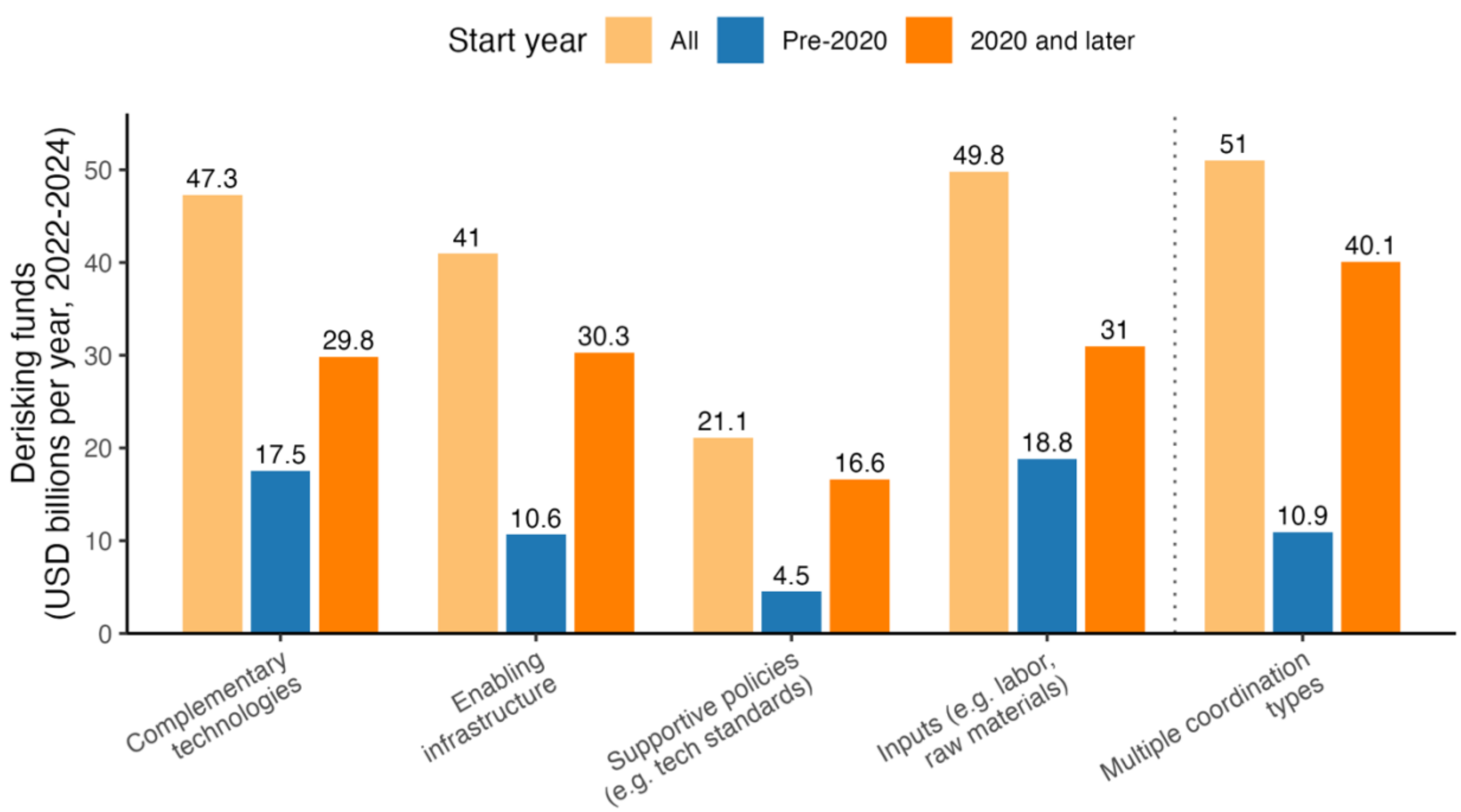

Supplementary Fig. 8. Percentage of derisking funds flowing through institutions performing each derisking type, separated by institution start year and color coded by derisking instrument, with technology-push disaggregated. Percentages are calculated within each start year group, using the sum of institutions' average annual derisking funds awarded from 2022–2024. Total derisking funds equal USD 91.07 billion across the full dataset of 122 institutions, USD 47.45 billion for institutions founded before 2020 (61 institutions), and USD 43.62 billion for institutions founded in 2020 or later (61 institutions). In this figure, technology-push is disaggregated into support for research, development, and demonstration (RD&D) and production. Production includes manufacturing, supply-chain infrastructure, and workforce development. Demand-pull derisking measures increase demand and facilitate deployment. The three categories of derisking are not mutually exclusive. For clarity, the figure is restricted to grants (orange), loans (blue), simultaneous use of grants and loans (yellow), and tax incentives (light green), as other derisking instruments (grey) account for only marginal funds. Source data are provided as a Source Data file.

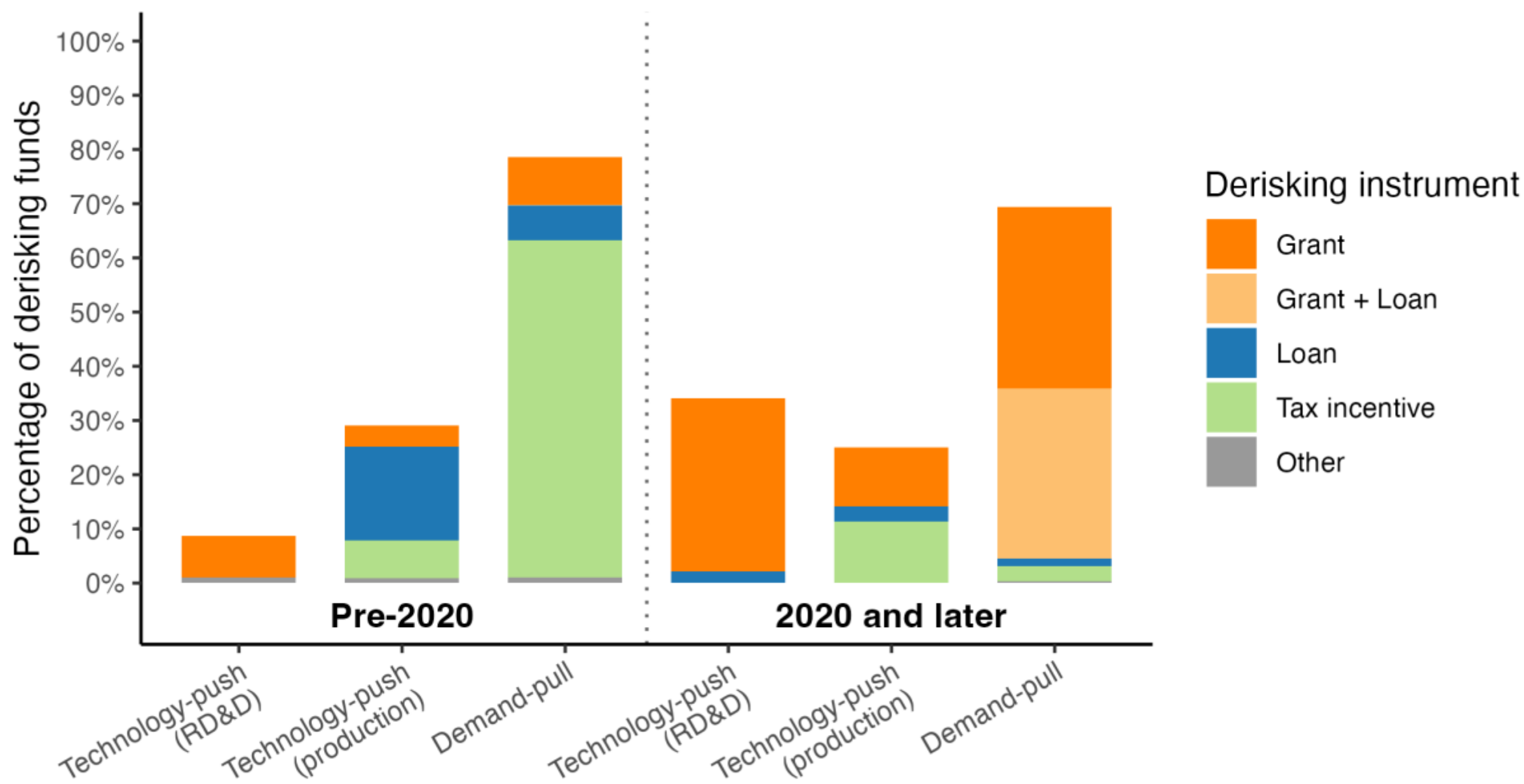

Supplementary Fig. 9. **a.** Percentage of institutions using different types of derisking instruments, and **b.** Percentage of institutions imposing different discipline types, color coded by institution start year. Yellow bars report the total percentage of institutions with each feature; blue bars report the percentage among institutions founded before 2020; and orange bars report the percentage among institutions founded in 2020 or later. The dataset includes 122 institutions: 61 founded before 2020 and 61 founded in 2020 or later. Neither derisking instruments nor discipline types are mutually exclusive. Environmental conditionalities impose requirements on project impacts beyond greenhouse gas emissions; greenhouse gas (GHG) emissions conditionalities favor lower-emissions projects; economic conditionalities impose requirements related to commercial potential or performance; and social equity conditionalities favor projects in low-income areas or areas more affected by the energy transition, or mandate labor standards or salary requirements. Checkpoints are points at which projects are evaluated so the institution can adjust course if necessary, including by clawing back funds. Direct involvement means that an institution retains some decision-making authority over its funded projects. Source data are provided as a Source Data file.

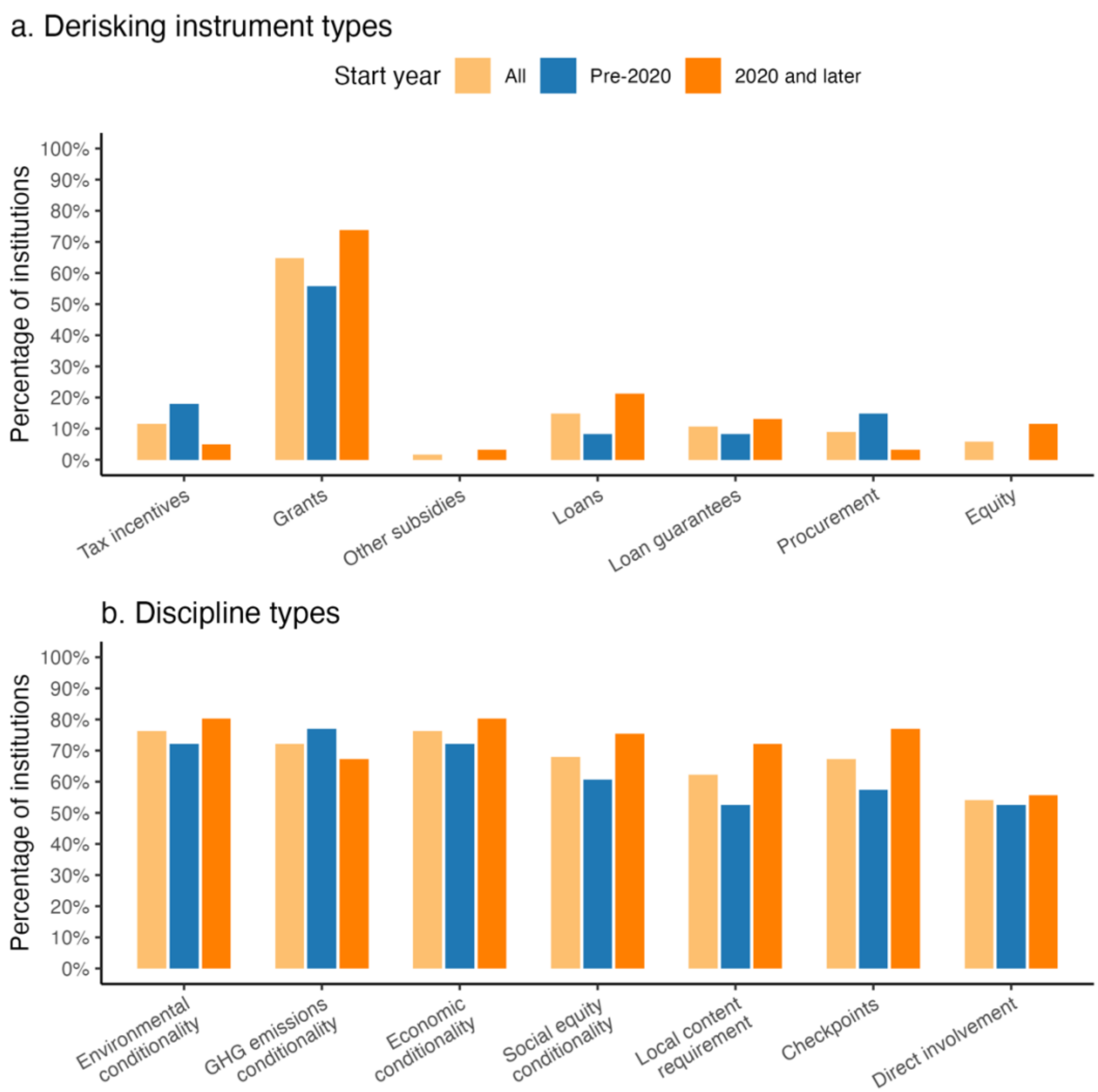

Supplementary Fig. 10. Percentage of derisking funds flowing through institutions using derisking instrument types, color coded by institution start year. Yellow bars report the percentage of total derisking funds flowing through institutions using a derisking instrument type; blue bars report the percentage among institutions founded before 2020; and orange bars report the percentage among institutions founded in 2020 or later. Derisking funds are measured as the sum of each institution's average annual funds awarded over 2022–2024. Total derisking funds equal USD 91.07 billion across the full dataset of 122 institutions, USD 47.45 billion for institutions founded before 2020 (61 institutions), and USD 43.62 billion for institutions founded in 2020 or later (61 institutions). Derisking instrument types are not mutually exclusive. For example, if an institution awards both grants and loans, the derisking funds associated with that institution will appear in both the "grants" and "loans" columns. Source data are provided as a Source Data file.

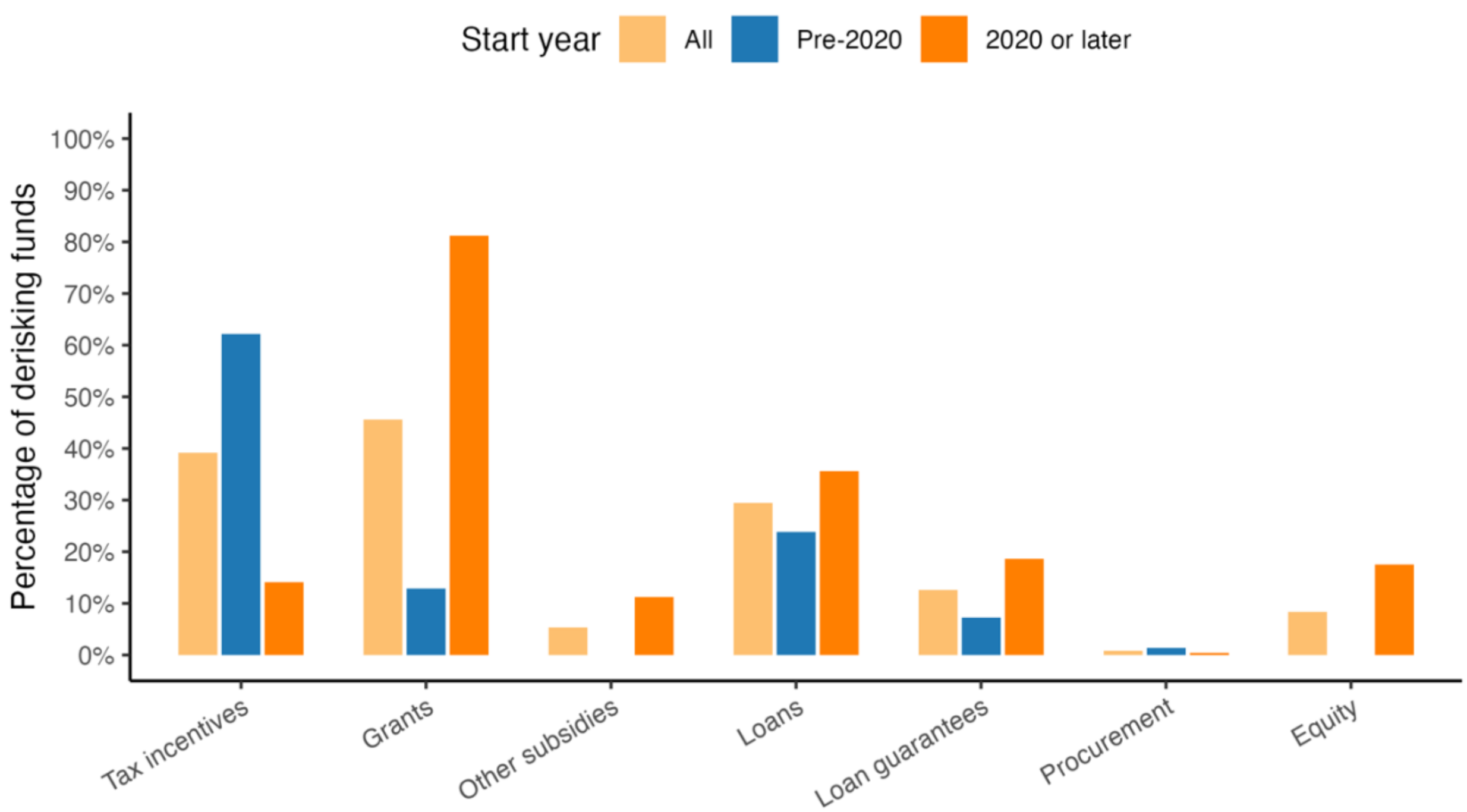

Supplementary Note 1. Institution Selection Criteria.

We review legislation relevant to US green and cleantech industrial and commercialization policy, US government spending data, and the websites of government departments to identify relevant institutions. We include all institutions that meet the following criteria 1-6 in the dataset.

Many institutions are nested in a hierarchy. That is, a parent institution may encompass multiple sub-institutions. We code the lowest level in the institutional hierarchy that meets our criteria for sample inclusion. We then code the next level(s) up (i.e., parent institutions) if they perform functions distinct from their sub-institutions and meet all other criteria for sample inclusion.

1. **Must focus on cleantech commercialization:** Does the institution dedicate at least 20% of its resources **OR** at least USD 50 million/year worth of resources to cleantech commercialization?
    a. **Percentage of resources** can be judged based on:
        i. % of budget dedicated to cleantech commercialization, **OR**
        ii. % of projects dedicated to cleantech commercialization, **OR**
        iii. % of focus areas dedicated to cleantech commercialization
    b. **Commercialization** refers to demonstration and deployment efforts involving technologies from TRL 4-10.
    c. **Cleantech** refers to technologies in the following categories, based on the IEA's ETP Clean Energy Technology Guide[1].
        i. Batteries and energy storage
        ii. Biofuels (liquid, solid, and biogas)
        iii. Carbon Capture, Utilization, and Storage (CCUS)
        iv. Buildings
        v. Grid optimization (e.g. grid efficiency upgrades, renewables integration, smart grids)
        vi. Hydrogen and fuel cells
        vii. Industry
        viii. Mobility (excluding batteries and biofuels, which are coded separately in their respective categories)
        ix. Nuclear (excluding generations I, II, or III; including small modular reactors)
        x. Renewables:
            1. Geothermal energy
            2. Hydroelectricity
            3. Ocean energy
            4. Solar energy
            5. Wind energy

    xi. Non-technology-specific: If an institution supports at least 10 of the 14 technologies listed above, or if it supports cleantech generally without imposing explicit limits on eligible technologies, we will code it as non-technology-specific.

  d. **Cleantech commercialization excludes:**
    i. Nuclear power generations I, II, or III
    ii. Natural climate solutions (e.g. offsets via tree planting)
    iii. Alternative proteins
    iv. Incumbent technologies (TRL 11)
    v. Fossil fuel-based heat and generation technologies
    vi. Institutions that exclusively focus on raw materials

2. **Must be an institution:** An institution is a program or organizational unit that shapes or manages commercialization activities.
  a. It shapes or manages activities if:
    i. There is dedicated staff**, OR**
    ii. The institution has published a strategic plan, annual report, or website that details its mission and approach, **OR**
    iii. The institution has a fiscal impact of over USD 100 million in funding.

3. **Must be a public US institution at the national level:**
  a. The US government established the institution, or collaborated with the private sector to create the institution, **OR**
  b. The US government provides at least 50% of the institution's funding.

4. **Must be current:**
  a. The institution must exist as of 2024.
  b. Note: If an institution has experienced changes in name and functions over time, we code the version that is current at the time of coding (2024).

5. **Must be relevant:**
  a. The institution meets one of the following criteria for relevance/importance based on its primary function:
    i. Primary goal setting or coordination function: always code
    ii. Primary derisking function: decide whether to code based on thresholds of minimum annual funds awarded according to the financial instrument used. These numbers refer to the amount of money dedicated to cleantech commercialization annually within the institution, not the institution's entire budget.
      1. Grants, other subsidies, and tax incentives: USD 10 million/year

        2. Loans, loan guarantees, and equity: USD 100 million/year
    iii. Primary external engagement function: has over 50 partners or network members
    iv. Note: primary discipline function does not exist within our sample (always accompanies other functions)
    v. Exceptions to relevance criteria may be granted in the following cases:
        1. Derisking institutions with disbursements below the thresholds are included if they provide direct commercialization support, such as accelerators.
        2. Derisking institutions that did not disburse funds during the 2022–2024 measurement period are included if their authorized budgets exceed the relevant threshold.
        3. Derisking institutions that operate primarily through procurement or loan guarantees are included even when disbursement amounts for 2022–2024 cannot be consistently recorded, as such data are often not reported in comparable form.

6. **Must have sufficient public materials to code:**
    a. We can only code institutions for which there is enough publicly available information to determine, with relative confidence, whether an institution performs the major functions and sub-variables in our dataset. Usually, one or more of the following sources will suffice:
        i. Detailed website
        ii. Annual report
        iii. Institution-level roadmap or strategic plan
        iv. Funding Opportunity Announcement, IRS Guidance, or Investment Strategy
        v. Secondary literature
    b. When we do not have sufficient material to code an institution that meets our other criteria for sample inclusion, we will instead code its parent institution (if one exists).

Supplementary Note 2. List of selected institutions, accompanied by acronym and abbreviation key. Names and hierarchy as of 2024. Institutions are listed hierarchically where applicable, beginning with the department or agency and followed by relevant sub-agencies, offices, initiatives, or programs.

Acronym and abbreviation key

AMMTO	Advanced Materials and Manufacturing Technologies Office
BTO	Building Technologies Office
DOC	Department of Commerce
DOD	Department of Defense
DOE	Department of Energy
DOI	Department of the Interior
DOT	Department of Transportation
EDA	Economic Development Administration
EERE	Office of Energy Efficiency and Renewable Energy
EPA	Environmental Protection Agency
FAA	Federal Aviation Administration
FECM	Office of Fossil Energy and Carbon Management
FHWA	Federal Highway Administration
FTA	Federal Transit Administration
GGRF	Greenhouse Gas Reduction Fund
GSA	General Services Administration
HUD	Department of Housing and Urban Development
IRS	Internal Revenue Service
LPO	Loan Programs Office
NREL	National Renewable Energy Laboratory
NSF	National Science Foundation
OCED	Office of Clean Energy Demonstrations
OE	Office of Electricity
OSC	Office of Strategic Capital
OTT	Office of Technology Transitions
SBA	Small Business Administration
SCEP	Office of State and Community Energy Programs
TCF	Technology Commercialization Fund
Treasury	U.S. Department of the Treasury
USDA	U.S. Department of Agriculture

List of institutions

1	Commercial Aviation Alternative Fuels Initiative (CAAFI)
2	DOC: EDA: Build Back Better Regional Challenge
3	DOC: EDA: Build to Scale (B2S)
4	DOC: EDA: Regional Technology and Innovation Hubs (Tech Hubs)
5	DOD: Air Force Office of Energy Assurance
6	DOD: Defense Innovation Unit (DIU)
7	DOD: Energy Security Technology Certification Program (ESTCP)
8	DOD: Office of Strategic Capital (OSC)
9	DOD: OSC: Credit Program
10	DOD: OSC and SBA: Small Business Investment Company Critical Technologies Initiative (SBICCT)
11	DOE and DOT: Joint Office of Energy and Transportation
12	DOE, DOT, and USDA: Sustainable Aviation Fuel Grand Challenge
13	DOE: Advanced Research Projects Agency–Energy (ARPA-E)
14	DOE: Cross-Cutting: Pathways to Commercial Liftoff Reports
15	DOE: Energy Earthshots Initiative
16	DOE: Energy Storage Grand Challenge
17	DOE: Federal Energy Management Program (FEMP)
18	DOE: Grid Deployment Office (GDO)
19	DOE: GDO: Grid Resilience and Innovative Partnerships (GRIP)
20	DOE: Loan Programs Office (LPO)
21	DOE: LPO: Advanced Technology Vehicles Manufacturing (ATVM) Loan Program
22	DOE: LPO: Title 17 Clean Energy Financing
23	DOE: LPO: Tribal Energy Finance Program
24	DOE: LPO and FECM: Carbon Dioxide Transportation Infrastructure Finance and Innovation Act (CIFIA) Funding
25	DOE: Manufacturing USA: Clean Energy Smart Manufacturing Innovation Institute (CESMII)
26	DOE: Manufacturing USA: Electrified Processes for Industry Without Carbon (EPIXC)
27	DOE: Manufacturing USA: Institute for Advanced Composites Manufacturing Innovation (IACMI)
28	DOE: Manufacturing USA: Rapid Advancement in Process Intensification Deployment Institute (RAPID)
29	DOE: Manufacturing USA: Reducing Embodied-Energy and Decreasing Emissions Institute (REMADE)
30	DOE: NREL: American-Made Challenges
31	DOE: NREL: Chevron Studio

32 DOE: NREL: Shell GameChanger Accelerator Powered by NREL (GCxN)
33 DOE: NREL: Wells Fargo Innovation Incubator
34 DOE: Office of Clean Energy Demonstrations (OCED)
35 DOE: OCED and Office of Nuclear Energy: Advanced Reactor Demonstration Projects
36 DOE: OCED: Carbon Capture Demonstration Projects Program
37 DOE: OCED: Carbon Capture Large-Scale Pilot Projects
38 DOE: OCED: Clean Energy Demonstration Program on Current and Former Mine Land
39 DOE: OCED: Distributed Energy Systems (DES) Demonstrations Program
40 DOE: OCED: Energy Improvement in Rural or Remote Areas (ERA)
41 DOE: OCED: Industrial Demonstrations Program
42 DOE: OCED: Long-Duration Energy Storage Demonstrations
43 DOE: OCED: Regional Clean Hydrogen Hubs (H2Hubs)
44 DOE: OCED: Regional Direct Air Capture Hubs (DAC)
45 DOE: Office of Electricity (OE): Energy Storage Division
46 DOE: Office of Energy Efficiency and Renewable Energy (EERE)
47 DOE: EERE: Advanced Materials and Manufacturing Technologies Office (AMMTO)
48 DOE: EERE: AMMTO: Lab-Embedded Entrepreneurship Program (LEEP)
49 DOE: EERE: Bioenergy Technologies Office (BETO)
50 DOE: EERE: Building Technologies Office (BTO)
51 DOE: EERE: BTO: Better Buildings Initiative
52 DOE: EERE: BTO: Zero Energy Ready Home Program
53 DOE: EERE: Geothermal Technologies Office (GTO)
54 DOE: EERE: Hydrogen and Fuel Cell Technologies Office (HFTO)
55 DOE: EERE: Industrial Efficiency and Decarbonization Office (IEDO)
56 DOE: EERE: Solar Energy Technologies Office (SETO)
57 DOE: EERE: Vehicle Technologies Office (VTO)
58 DOE: EERE: VTO: Clean Cities and Communities Initiative
59 DOE: EERE: Water Power Technologies Office (WPTO)
60 DOE: EERE: Wind Energy Technologies Office (WETO)
61 DOE: Office of Fossil Energy and Carbon Management (FECM)
62 DOE: FECM: CarbonSAFE Initiative
63 DOE: Office of Indian Energy Policy and Programs
64 DOE: Office of Manufacturing and Energy Supply Chains (MESC)
65 DOE: Office of Science: Small Business Innovation Research and Small Business Technology Transfer Programs Office (SBIR/STTR Programs Office)
66 DOE: Office of State and Community Energy Programs (SCEP)
67 DOE: SCEP: Community Energy Programs
68 DOE: SCEP: Energy Efficiency and Conservation Block Grant

69 DOE: SCEP: Energy Efficiency Revolving Loan Funds
70 DOE: SCEP: Renew America's Schools
71 DOE: SCEP: State and Local Energy Efficiency Action Network (SEE Action)
72 DOE: SCEP: State Energy Program
73 DOE: SCEP: Technical Assistance for the Adoption of Building Codes
74 DOE: SCEP: Weatherization Assistance Program
75 DOE: Office of Technology Transitions (OTT)
76 DOE: OTT: Energy I-Corps
77 DOE: OTT: Foundation for Energy Security and Innovation (FESI)
78 DOE: OTT: Technology Commercialization Fund (TCF)
79 DOE: OTT and OCED: TCF: Collaborative Alignment for Critical Technology Industries (CACTI)
80 DOE: OTT, OCED, FECM, and EERE: TCF: Voucher Program
81 DOI: Office of Indian Affairs: Tribal Electrification Program
82 DOT: FAA: Continuous Lower Energy, Emissions, and Noise (CLEEN) Program
83 DOT: FAA: Fueling Aviation's Sustainable Transition (FAST) Grants
84 DOT: FHWA: Carbon Reduction Program (CRP)
85 DOT: FHWA: Charging and Fueling Infrastructure Grant Program
86 DOT: FHWA: Low-Carbon Transportation Materials Grants Program
87 DOT: FHWA: National Electric Vehicle Infrastructure (NEVI) Program
88 DOT: FTA: Electric or Low-Emitting Ferry Pilot Program
89 DOT: FTA: Low or No Emission Grant Program
90 EPA: Clean Heavy-Duty Vehicles Program
91 EPA: Clean School Bus Program
92 EPA: Diesel Emissions Reduction Act (DERA) Funding
93 EPA: ENERGY STAR
94 EPA: Greenhouse Gas Reduction Fund (GGRF)
95 EPA: GGRF: Clean Communities Investment Accelerator
96 EPA: GGRF: National Clean Investment Fund
97 EPA: GGRF: Solar for All
98 GSA: Center for Emerging Building Technologies
99 GSA: Low Embodied Carbon Program
100 HUD: Green and Resilient Retrofit Program
101 NSF: Regional Innovation Engines
102 Treasury: IRS: Advanced Energy Project Credit (48C)
103 Treasury: IRS: Advanced Manufacturing Production Credit (45X)
104 Treasury: IRS: Alternative Fuel Vehicle Refueling Property Credit (30C)
105 Treasury: IRS: Clean Hydrogen Production Tax Credit (45V)

106 Treasury: IRS: Clean Vehicle Credit (30D)
107 Treasury: IRS: Credit for Carbon Oxide Sequestration (45Q)
108 Treasury: IRS: Credit for Qualified Commercial Clean Vehicles (45W)
109 Treasury: IRS: Energy Efficiency Home Improvement Credit (25C)
110 Treasury: IRS: Energy Efficient Commercial Buildings Deduction (179D)
111 Treasury: IRS: Investment Tax Credit for Energy Property (48)
112 Treasury: IRS: New Energy Efficient Homes Credit (45L)
113 Treasury: IRS: Production Tax Credit for Electricity from Renewables (45)
114 Treasury: IRS: Residential Clean Energy Credit (25D)
115 Treasury: IRS: Tax Credits for Biodiesel and Renewable Diesel (40A)
116 USDA: Rural Development: BioPreferred Program
117 USDA: Rural Development: Biorefinery, Renewable Chemical, and Biobased Product Manufacturing Assistance Program
118 USDA: Rural Development: Empowering Rural America (New ERA)
119 USDA: Rural Development: Higher Blends Infrastructure Incentive Program
120 USDA: Rural Development: Powering Affordable Clean Energy (PACE) Program
121 USDA: Rural Development: Rural Energy for America Program
122 USDA: Rural Development: Rural Energy Savings Program

Supplementary Table 1. Full coding scheme.

**1: Background Information Variables**		
**Name**	**Description**	**Sources consulted**
	1.1: Basic Information Variables	
institution_name	Text input: Institution name.	Institution website or annual report.
description	Text input: Brief 1-2 sentence description of the institution's work.	Institution website or annual report.
parent_institution	Binary indicator: Institution manages another institution in the dataset. (1 = true, 0 = false) If so, in the coding, we do not attribute any of the sub-institution's characteristics to the parent. Only functions that are specifically performed at the parent institution level will be coded.	Institution website or annual report indicates the relationship between different institutions. If we decide to code sub-institutions (based on the criteria for sample inclusion), then we will code 1 for parent_institution.
	1.2: Policy Mission Variables	
M_competitiveness_growth	Binary indicator: Institution references competitiveness and economic growth in its mission statement or as one of its major objectives. (1 = true, 0 = false)	Institution's mission statement or major objectives, found on the institution's website or in an annual report.
M_energy_security	Binary indicator: Institution references energy security in its mission statement or as one of its major objectives. (1 = true, 0 = false)	
M_climate_change	Binary indicator: Institution references climate change mitigation in its mission statement or as one of its major objectives. (1 = true, 0 = false)	
M_soc_equity	Binary indicator: Institution references social equity in its mission statement or as one of its major objectives. (1 = true, 0 = false)	
	1.3: Institutional Change Variables	
start_year	Numeric input: Year the institution was founded.	Institution website or annual report. Usually described in the "about us" or "history" section of the website, or in the "news" section as a press release.
end_year	Numeric input: Year the institution was discontinued (if applicable).	
org_change	Text input: Notes on any major organizational changes observed since the institution's founding, such as a name change, merger, split, change in parent institution, etc. (if applicable).	
	1.4: Technology Focus Variables	
batteries_storage	Binary indicator: Institution has a focus on batteries or energy storage. (1 = true, 0 = false)	Institution website or annual report. Usually in the "about us" section or on a "portfolio" page listing funded projects. May also be found via Funding Opportunity Announcements and Investment Policy Statements that provide a list of eligible technologies. Technology categories are not mutually exclusive.
CCUS	Binary indicator: Institution has a focus on carbon capture, utilization, and storage. Includes carbon transport. (1 = true, 0 = false)	
buildings	Binary indicator: Institution has a focus on energy efficiency in buildings construction and renovation, cooking, heating and cooling, lighting, or systems integration. (1 = true, 0 = false)	
grid_optimization	Binary indicator: Institution has a focus on grid technologies and upgrades that can facilitate the integration of renewables or increase energy efficiency in transmission and distribution. Also includes technologies such as distributed energy systems, microgrids with cleantech integration, smart grids, and virtual power plants. (1 = true, 0 = false)	
hydrogen	Binary indicator: Institution has a focus on hydrogen and fuel cells. (1 = true, 0 = false)	
industry	Binary indicator: Institution has a focus on technologies that reduce emissions associated with the production of industrial materials such as aluminum, cement and concrete, chemicals and plastics, iron and steel, and pulp and paper. Also includes technologies that reduce emissions associated with industrial heating and technologies	

	associated with e-waste and metals recycling. (1 = true, 0 = false)	
mobility	Binary indicator: Institution has a focus on technologies that reduce emissions in aviation, rail, road transport, and shipping. This technology group is restricted to the vehicles/aircraft/vessels and their components, as well as charging and refueling technologies. However, batteries and fuels (including hydrogen and biofuels) are coded as separate technology areas. (1 = true, 0 = false)	
nuclear	Binary indicator: Institution has a focus on generation IV nuclear reactors or small modular reactors. (1 = true, 0 = false) Institutions that focus solely on generations I, II, and III are excluded from the dataset.	
biofuels	Binary indicator: Institution has a focus on liquid biofuels, solid biofuels, or biogases. (1 = true, 0 = false)	
geothermal	Binary indicator: Institution has a focus on geothermal technologies. (1 = true, 0 = false)	
hydro	Binary indicator: Institution has a focus on hydropower technologies. (1 = true, 0 = false)	
ocean	Binary indicator: Institution has a focus on ocean energy technologies. (1 = true, 0 = false)	
solar	Binary indicator: Institution has a focus on solar energy technologies. (1 = true, 0 = false)	
wind	Binary indicator: Institution has a focus on wind energy technologies. (1 = true, 0 = false)	
not_tech_specific	Binary indicator: Institution either does not specify a technological focus, has projects in 10 or more technology areas as defined above, or has a cross-cutting focus such as increasing clean energy manufacturing capacity or supporting the clean energy innovation ecosystem. (1 = true, 0 = false)	
	1.5: Innovation Process Variables	
IP_research	Binary indicator: Institution supports basic research, TRL 1. (1 = true, 0 = false) Institutions that focus exclusively on basic research are excluded from the dataset, but institutions that support basic research as part of a broader portfolio are recorded here.	Institution website or annual report. Usually in the “about us” section or on a “portfolio” page listing funded projects. May also be found via Funding Opportunity Announcements and Investment Policy Statements that include eligible project types, including TRL levels.
IP_development	Binary indicator: Institution supports technologies in the development stage, TRL 2-5. (1 = true, 0 = false) Institutions that focus exclusively on early-stage development (TRL 2-3) are excluded from the dataset, but institutions that support technologies at TRL 4-5, or institutions that support earlier TRLS as part of a broader portfolio, are recorded here.	
IP_demonstration	Binary indicator: Institution supports technologies in the demonstration stage, TRL 6-8. (1 = true, 0 = false)	
IP_deployment	Binary indicator: Institution supports technologies in the deployment stage, TRL 9-10. (1 = true, 0 = false)	
IP_diffusion	Binary indicator: Institution supports technologies in the diffusion stage, TRL 11. (1 = true, 0 = false) Institutions that exclusively support technologies at the diffusion stage are excluded from the dataset, but institutions that support diffusion as part of a broader portfolio are recorded here.	
	1.6: Oversight Variables	
O_commerce	Binary indicator: Institution is overseen by the Department of Commerce. (1 = true, 0 = false)	Institution website or annual report. Usually in the “about us” section.
O_irs	Binary indicator: Institution is overseen by the Internal Revenue Service. (1 = true, 0 = false)	
O_interior	Binary indicator: Institution is overseen by the Department of the Interior. (1 = true, 0 = false)	
O_gsa	Binary indicator: Institution is overseen by the General Services Administration. (1 = true, 0 = false)	
O_defense	Binary indicator: Institution is overseen by the Department of Defense (now called the Department	

	of War, but was the Department of Defense at the time of coding). (1 = true, 0 = false)	
O_nsf	Binary indicator: Institution is overseen by the National Science Foundation. (1 = true, 0 = false)	
O_energy	Binary indicator: Institution is overseen by the Department of Energy. (1 = true, 0 = false)	
O_environment	Binary indicator: Institution is overseen by the Environmental Protection Agency. (1 = true, 0 = false)	
O_transport	Binary indicator: Institution is overseen by the Department of Transport. (1 = true, 0 = false)	
O_agriculture	Binary indicator: Institution is overseen by the United States Department of Agriculture. (1 = true, 0 = false)	
O_housing	Binary indicator: Institution is overseen by the Department of Housing and Urban Development. (1 = true, 0 = false)	
O_independent	Binary indicator: Institution is a public-private partnership that is primarily overseen by the private sector. (1 = true, 0 = false)	
1.7: Political Conflict and Institutional Learning Variables		
F_evaluation	Binary indicator: Institution regularly monitors and evaluates its programs. (1 = true, 0 = false)	Record of monitoring and evaluation on the institution's website, its parent institution's website, or the Government Accountability Office.
appointment	Binary indicator: Leader of this institution is politically appointed (1) or a career bureaucrat (0).	Office of Personnel Management Periodically Listing Updates to Management (PLUM) data. Supplementary information found via press releases on hiring/appointment decisions.
conflict	Text input: Notes on any evidence of political conflict the institution has encountered.	News articles, academic literature, grey literature.
**2: Function: Goal setting**		
F_goal	Binary indicator: Institution engages in goal setting activities. (1 = true, 0 = false) This variable will be equal to one if the institution engages in any strategy development, modeling, or analysis, or if it has set any *specific* goals *itself* (rather than inheriting them).	Input depends on the results of the other goal setting variables, detailed below.
2.1: Roadmapping, Modeling, and Analysis Variables		
SP_since_2020	Binary indicator: Institution has published at least one strategy document, such as a roadmap or multi-year program plan, between 2020-2024. (1= yes, 0 = no)	Institution website, usually in a section titled "documents," "research," or "reports."
SP_project_support	Binary indicator: Institution supports and monitors planning activities at the project level. (1 = true, 0 = false) This is often done through phased grants, in which phase one is dedicated to strategic planning. Based on these plans, the institution then selects institutions for phase two implementation grants.	Institution website, annual report, or Funding Opportunity Announcements.
Modeling_analysis_div	Binary indicator: Institution has a division dedicated to research, modeling, and analysis relevant to the commercialization of clean energy technologies. (1 = true, 0 = false)	Institution website or annual report. A division dedicated to research, analysis, and modeling will usually be referenced as part of the institution's organizational structure and may have an entire section of the website or annual report listing research outputs.
SP_scope	Text input: Scope of strategic planning activities. Input options: project-specific, institution-specific, single tech-specific, multiple tech-specific, sector-wide, economy-wide. If there is evidence for more than one scope (e.g. an institution performs strategic planning at both an institution-specific level and a sector-wide level), we record the broadest type.	Institution roadmapping documents, research outputs, and/or Funding Opportunity Announcements (for institutions performing project-level roadmapping).
2.2: Emissions Goal Variables		
GT_emissions	Binary indicator: Institution sets or references a goal to reduce emissions. (1 = true, 0 = false)	Institution website, roadmap, or work plan. Goals must be explicitly communicated to warrant coding. That is, we do not code for goals that are merely implied by an institution's projects.
Em_specificity	Text input: Specificity of emissions goal. Input options: general, specific. For a goal to be specific, it must be measurable and time-bound.	
Em_origin	Text input: Origin of emissions goal. Input options: self, outside institution, legislature, national leader.	

Em_scope	Text input: Scope of emissions goal. Input options: single tech-specific, multiple tech-specific, sector-wide, economy-wide.	
Em_context	Text input: Context of emissions goal. Input options: within roadmap, standalone, inherited.	
2.3: Sustainability Goal Variables		
GT_sustainability	Binary indicator: Institution sets or references a goal to increase sustainability associated with cleantech. (1 = true, 0 = false) Examples of sustainability goals include goals relevant to energy efficiency, recyclability, decreasing resource use, or ethical sourcing of raw materials.	Institution website, roadmap, or work plan. Goals must be explicitly communicated to warrant coding. That is, we do not code for goals that are merely implied by an institution's projects.
Sus_specificity	Text input: Specificity of sustainability goal. Input options: general, specific. For a goal to be specific, it must be measurable and time-bound.	
Sus_origin	Text input: Origin of sustainability goal. Input options: self, outside institution, legislature, national leader.	
Sus_scope	Text input: Scope of sustainability goal. Input options: single tech-specific, multiple tech-specific, sector-wide, economy-wide.	
Sus_context	Text input: Context of sustainability goal. Input options: within roadmap, standalone, inherited.	
2.4: Cost Reduction Goal Variables		
GT_cost	Binary indicator: Institution sets or references a goal to decrease the costs associated with cleantech. (1 = true, 0 = false)	Institution website, roadmap, or work plan. Goals must be explicitly communicated to warrant coding. That is, we do not code for goals that are merely implied by an institution's projects.
Cost_specificity	Text input: Specificity of cost goal. Input options: general, specific. For a goal to be specific, it must be measurable and time-bound.	
Cost_origin	Text input: Origin of cost goal. Input options: self, outside institution, legislature, national leader.	
Cost_scope	Text input: Scope of cost goal. Input options: single tech-specific, multiple tech-specific, sector-wide, economy-wide.	
Cost_context	Text input: context of cost goal. Input options: within roadmap, standalone, inherited.	
2.5: Deployment Goal Variables		
GT_deployment	Binary indicator: Institution sets or references a goal to increase deployment of cleantech. (1 = true, 0 = false)	Institution website, roadmap, or work plan. Goals must be explicitly communicated to warrant coding. That is, we do not code for goals that are merely implied by an institution's projects.
Dep_specificity	Text input: Specificity of deployment goal. Input options: general, specific. For a goal to be specific, it must be measurable and time-bound.	
Dep_origin	Text input: Origin of deployment goal. Input options: self, outside institution, legislature, national leader.	
Dep_scope	Text input: Scope of deployment goal. Input options: single tech-specific, multiple tech-specific, sector-wide, economy-wide.	
Dep_context	Text input: Context of deployment goal. Input options: within roadmap, standalone, inherited.	
2.6: Supply Chain Goal Variables		
GT_supply_chain	Binary indicator: Institution sets or references goals relevant to building a domestic supply chain or boosting innovation/commercialization ecosystems for cleantech. (1 = true, 0 = false). Examples of supply chain goals include establishing a secure supply of critical minerals, building up manufacturing capacity for clean energy technologies, or training a workforce to operate or invent clean energy technologies.	Institution website, roadmap, or work plan. Goals must be explicitly communicated to warrant coding. That is, we do not code for goals that are merely implied by an institution's projects.
Sup_specificity	Text input: Specificity of supply chain goal. Input options: general, specific. For a goal to be specific, it must be measurable and time-bound.	
Sup_origin	Text input: Origin of supply chain goal. Input options: self, outside institution, legislature, national leader.	
Sup_scope	Text input: Scope of supply chain goal. Input options: single tech-specific, multiple tech-specific, sector-wide, economy-wide.	

Sup_context	Text input: Context of supply chain goal. Input options: within roadmap, standalone, inherited.	
**3: Function: Coordination**		
F_coordination	Binary indicator: Institution engages in coordination activities. (1 = true, 0 = false) We define coordination as reducing bottlenecks in cleantech commercialization through actions in one of the four categories below. Institutions that solely engage in coordination are excluded from the dataset (e.g. institutions that only focus on raw material sourcing or only develop innovative financial instruments to support cleantech). Institutions that engage in coordination while also directly supporting one of the technologies listed in section 1.4 are included in the dataset.	Input depends on the results of the other coordination variables, detailed below.
C_tech	Institution coordinates the development of complementary technologies. (1 = true, 0 = false)	Any source that gives an overview of the institution's primary program areas. This can primarily be found on the institution website or annual report. We also reference Funding Opportunity Announcements when the website and annual report are not sufficiently detailed.
C_infrastructure	Institution coordinates the development of enabling infrastructure and cleantech (e.g. charging infrastructure and EVs, energy storage infrastructure and renewables, upgraded grid infrastructure and renewables). (1 = true, 0 = false)	
C_policies	Institution coordinates the development of policies to support cleantech commercialization (e.g. permitting reform for energy infrastructure, technology standards, regulatory sandboxes). (1 = true, 0 = false)	
C_inputs	Institution coordinates the necessary inputs to support cleantech commercialization (e.g. reskilling or upskilling programs, programs in critical minerals sourcing).	
**4: Function: Derisking**		
F_derisking	Binary indicator: Institution engages in derisking activities. (1 = true, 0 = false) We define derisking as providing financial or in-kind support to decrease technology and market risk.	Input depends on the results of the other derisking variables, detailed below.
4.1: Derisking Amount Variables		
De_2022	Numeric input: Amount of derisking funds awarded in 2022 through grants, loans, and tax credits.	For grants and loans, awarded funds per year found through 1) database of funds awarded on institution website, 2) annual report recording the total funds awarded in a year, or 3) press releases announcing awardees and award amounts from institution website. In select cases when there is no data available for funds awarded in a given year, we estimate based on award amounts in prior years or information about the total pot of money budgeted over a time period (e.g. USD 100 million to be distributed over 5 years is estimated at USD 20 million/yr).<br><br>For tax credits, we rely on Estimates of Federal Tax Expenditures from the Joint Committee on Taxation[2–4]. For each year's data, we rely on the Estimates of Federal Tax Expenditures report published in that year (e.g. De_2024 is informed by the Estimates of Federal Tax Expenditures for Fiscal Years 2024-2028, published in December 2024). For certain tax credits in select years, the IRS has released data on actual tax credits claimed (as opposed to expenditure estimates)[5]. In these cases, we use the IRS data. Finally, for tax credits that do not have available data through the Joint Committee on Taxation or the IRS, we use estimates from the Office of Management and Budget, prepared by the Treasury's Office of Tax
De_2023	Numeric input: Amount of derisking funds awarded in 2023 through grants, loans, and tax credits.	
De_2024	Numeric input: Amount of derisking funds awarded in 2024 through grants, loans, and tax credits.	
De_avg	Numeric input: Average amount of derisking funds awarded annually 2022-2024 through grants, loans, and tax credits.	

		Analysis[6–8]. For tax credits without available estimates from any of these sources, we exclude them from the dataset.
	4.2: Derisking Type Variables	
De_tech_push_RDD	Binary indicator: Institution engages in technology-push derisking through financial assistance or in-kind support for RD&D. (1 = true, 0 = false)	Institution website or annual report. Usually in the "about us" section or on a "portfolio" page listing funded projects. May also be found via Funding Opportunity Announcements and Investment Policy Statements that include eligible project types.
De_tech_push_production	Binary indicator: Institution engages in technology-push derisking through financial assistance for manufacturing, supply-chain infrastructure, or workforce development. (1 = true, 0 = false)	
De_demand_pull	Binary indicator: Institution engages in demand-pull derisking measures, such as tax credits and rebates for consumers or government procurement. (1= true, 0 = false)	
De_tech	Binary indicator: Institution engages in technology derisking measures, meaning that it addresses risk associated with technology performance and cost. (1 = true, 0 = false)	
De_market	Binary indicator: Institution engages in market derisking measures, meaning that it addresses risk associated with large-scale production or construction. (1= true, 0 = false)	
	4.3: Derisking Instrument Variables	
De_tax_incentive	Binary indicator: Institution uses tax incentives to derisk. (1 = true, 0 = false)	Institution website or annual report. Usually in the "about us" section or on a "portfolio" page listing funded projects and the financial instruments used to support them. May also be found via Funding Opportunity Announcements listing available funding types.
De_grant	Binary indicator: Institution uses grants to derisk. (1 = true, 0 = false)	
De_cost_share_grant	Binary indicator: Institution uses grants to derisk, and grants have a cost-share requirement. That is, grants will provide a portion of the funding and require projects to obtain additional funding from other sources. (1 = true, 0 = false) If true, this institution would have a 1 for both De_grant and De_cost_share_grant.	
De_other_subsidy	Binary indicator: Institution uses subsidies besides grants to derisk. (1 = true, 0 = false)	
De_loan	Binary indicator: Institution uses loans to derisk. (1 = true, 0 = false)	
De_cost_share_loan	Binary indicator: Institution uses loans to derisk and requires projects to obtain additional funding from other sources. (1 = true, 0 = false) If true, this institution would have a 1 for both De_loan and De_cost_share_loan.	
De_loan_guarantee	Binary indicator: Institution uses loan guarantees to derisk. (1 = true, 0 = false)	
De_procurement	Binary indicator: Institution uses government procurement to derisk. (1 = true, 0 = false)	
De_equity	Binary indicator: Institution uses direct equity to derisk. A Simple Agreement for Future Equity (SAFE) would also fall under this category. (1 = true, 0 = false)	
De_other	Text input: Notes on any other instruments or methods the institution uses to derisk.	
	**5: Function: Discipline**	
F_discipline	Binary indicator: Institution imposes discipline. (1 = true, 0 = false)	Input depends on the results of the other discipline variables, detailed below.
Di_environment	Binary indicator: Institution imposes an environmental conditionality, meaning that it has requirements for reducing environmental harm or promoting environmental benefit. Required NEPA reviews also fall under this category. (1 = true, 0 = false)	Funding Opportunity Announcements, Investment Policy Statements, tax credit guidance from the IRS, and any other documents that detail funding requirements and application review processes.
Di_emissions	Binary indicator: Institution has specific requirements for limiting or reducing greenhouse gas (GHG)	

	emissions in order to be considered or prioritized for funding. (1 = true, 0 = false)	
Di_economic	Binary indicator: Institution has specific requirements for proving an institution's potential for economic success (e.g. cleantech cost reduction, commercialization potential, profit) in order to be considered or prioritized for funding. (1 = true, 0 = false)	
Di_soc_equity	Binary indicator: Institution has specific requirements for a project to increase social equity in order to be considered or prioritized for funding. For example, bonuses for meeting wage and apprenticeship requirements, locating in an energy community, or locating in an economically disadvantaged area. (1 = true, 0 = false)	
Di_local_content	Binary indicator: Institution requires, or offers additional benefits for, projects with components mined or produced domestically, or projects employing US citizens or US firms. (1 = true, 0 = false)	
Di_checkpoint	Binary indicator: Institution requires projects to meet specific checkpoints, also known as go/no-go decision points, in order to continue receiving support. (1 = true, 0 = false)	
Di_direct_involvement	Binary indicator: Institution maintains significant involvement (including decision-making influence) over funded projects. In the US case, this is usually done through a Cooperative Agreement award structure. (1 = true, 0 = false)	
**6: Function: External Engagement**		
F_external_engagement	Binary indicator: Institution engages in external engagement. (1 = true, 0 = false)	Input depends on the results of the other external engagement variables, detailed below.
6.1: External Engagement Target Group Variables		
EE_firms	Binary indicator: Institution engages with firms through one of the external engagement methods listed below in section 6.1, apart from ad hoc consultation. (1 = true, 0 = false)	Institution website and annual report, with special attention to "news," "events" and "leadership" sections. We do not record actors engaged solely in ad hoc consultation because it can be open to anyone and the consulted parties are often not explicitly listed.
EE_investors	Binary indicator: Institution engages with investors through one of the external engagement methods listed below in section 6.1, apart from ad hoc consultation. (1 = true, 0 = false)	
EE_nonprofits	Binary indicator: Institution engages with nonprofits through one of the external engagement methods listed below in section 6.1, apart from ad hoc consultation. (1 = true, 0 = false)	
EE_research_inst	Binary indicator: Institution engages with research institutions (e.g. universities, think tanks, national labs) through one of the external engagement methods listed below in section 6.1, apart from ad hoc consultation. (1 = true, 0 = false)	
EE_communities	Binary indicator: Institution engages with communities through one of the external engagement methods listed below in section 6.1, apart from ad hoc consultation. "Communities" refers to organizations or groups representing the interests of people in locations hosting clean energy projects. (1 = true, 0 = false)	
EE_citizens	Binary indicator: Institution engages with citizens through one of the external engagement methods listed below in section 6.1, apart from ad hoc consultation. Citizens are distinguished from communities because engagement efforts are not tied to a specific place. For example, general household outreach aimed at increasing awareness of renewable energy options would fall under this category. (1 = true, 0 = false)	

EE_congress	Binary indicator: Institution engages with Congress through one of the external engagement methods listed below in section 6.1, apart from ad hoc consultation. We code for external engagement with Congress only when a dedicated team, liaison, or event for Congressional engagement is referenced in an institution's materials. We do not include Congressional hearings or informal/unpublished negotiations. (1 = true, 0 = false)	
EE_subnational_gov	Binary indicator: Institution engages with subnational governments through one of the external engagement methods listed below in section 6.1, apart from ad hoc consultation. (1 = true, 0 = false)	
EE_other_inst	Binary indicator: Institution engages with other public institutions through one of the external engagement methods listed below in section 6.1, apart from ad hoc consultation. (1 = true, 0 = false)	
6.2: External Engagement Type Variables		
EE_consultation_adhoc	Binary indicator: Institution engages with outside actors through ad hoc consultation, such as through Requests for Information (RFIs) or listening sessions. (1 = true, 0 = false)	Institution website and annual report, with special attention to "news," "events" and "leadership" sections. For EE_consultation_adhoc, we search the internet for evidence of RFIs and listening sessions. We also examine the institution's roadmaps for evidence of stakeholder input.
EE_advisory_committee	Binary indicator: Institution engages with outside actors through a sub-institution designed to facilitate regular consultation, such as an advisory committee. (1 = true, 0 = false)	
EE_comanagement	Binary indicator: Institution engages with outside actors by co-managing institution funds or activities. For example, the institution may have a board of directors including members of the private sector. Another form of co-management occurs when an institution awards funds to intermediaries such as states or financial institutions, which then make sub-awards. (1 = true, 0 = false)	
EE_contracting_outsourcing	Binary indicator: Institution engages with outside actors by contracting or outsourcing some components of program delivery, such as technical assistance or research and monitoring. (1 = true, 0 = false)	
EE_support	Binary indicator: Institution engages with outside actors by providing direct support, such as through technical assistance, mentorship, or use of national lab facilities. (1 = true, 0 = false)	
EE_conferences_events	Binary indicator: Institution engages with outside actors by hosting conferences and events. (1 = true, 0 = false)	
6.3: External Engagement Network Characteristic Variables		
EE_one_on_one	Binary indicator: Institution performs external engagement on a one-on-one basis with outside actors. (1 = true, 0 = false)	Institution website, annual report, and Funding Opportunity Announcements.
EE_multiple_same	Binary indicator: Institution's external engagement approach creates a network of multiple actors of the same type. For example, a forum where cities can share knowledge and support about their decarbonization efforts. (1 = true, 0 = false)	
EE_multiple_different	Binary indicator: Institution's external engagement approach creates a network of multiple actors of different types. For example, an advisory committee designed to bring together firms from across the supply chain. (1 = true, 0 = false)	
EE_place_based	Binary indicator: Institution's work is place-based, meaning that programs target a specific region for development, demonstration, or deployment and tailor initiatives to local conditions at a systems level. (1 = true, 0 = false)	
EE_direct_networking	Binary indicator: Institution creates networks itself. In other words, the institution is the one directly connecting different outside actors. (1 = true, 0 = false)	

EE_outsourced_networking	Binary indicator: Institution outsources network creation to the outside actors. The most common mechanism for this is that the institution requires engagement with outside actors (such as community organizations and labor organizations) or requires projects to form consortia as a stipulation for funding. (1 = true, 0 = false)	

Supplementary Table 2. The second Trump Administration's strategies of institutional change.

Institution	Retrenching	Redesigning	Repurposing	Maintaining
Office of Clean Energy Demonstrations (OCED)	USD 0 budget request for FY2026; USD 12 billion in awards canceled with more rumored; 77% of staff resigned under deferred resignation program[2]	OCED mostly absorbed by the Office of Critical Minerals and Energy Innovation (CMEI), with some former OCED programs shifted to other parts of the DOE[9]	Second Trump administration will use OCED to manage USD 525 million in coal investments[10]	Advanced nuclear and critical minerals projects did not experience cuts[11]
Loan Programs Office (LPO)	Froze progress on USD 30 billion in conditional commitments; Three leadership changes in six months[12]; At least 50% of staff resigned under deferred resignation program[12]	Energy Infrastructure Reinvestment Program rebranded as the Energy Dominance Financing Program, canceled emissions reduction requirements[13]; broader LPO also renamed the Office of Energy Dominance Financing[14]	Executive Order on May 23, 2025 instructed the LPO to prioritize activities that support nuclear energy[15]	Finalized a loan guarantee to American Electric Power for transmission upgrades (conditional commitment made under Biden)[16]
Office of Energy Efficiency and Renewable Energy (EERE)	DOE canceled 321 awards worth up to USD 7.5 billion in October 2025, and 200 of these awards were funded by EERE[17]; DOE FY2026 budget request reflects a decrease of 74% compared to the 2025 budget[18]	EERE absorbed by CMEI, leading to a reorganization of its institutional structure[9]	President's FY2026 budget request promises to pivot away from "climate change-related activities" and support "technologies that promote firm baseload power...such as bioenergy"[19]	
National Electric Vehicle Infrastructure (NEVI) program		Reduced application requirements under the second Trump administration. Transportation Secretary Sean P. Duffy identified this as a strategy to "cut waste"[20]		After an initial funding freeze, the Department of Transportation concluded that it could not cancel NEVI[21]
Clean Energy Tax Credits that were established or renewed by the IRA	One Big Beautiful Bill Act (OBBBA) mandates early phaseout of: Technology Neutral Clean Electricity Credits (for solar and wind), EV Credits, Used EV Credit, Business Clean Vehicles Credit, Alternative Fuel Vehicle Refueling Property Credit, Energy Efficient Home Credit, Clean Hydrogen Credit, Residential Clean Electricity Credit, Residential Energy Efficiency Home Improvement Credit, Energy Efficient Commercial Buildings Deduction, Advanced Manufacturing Credit (for wind components and critical minerals)[22]	OBBBA applies stricter Foreign Entity of Concern restrictions for: the Existing Nuclear Credit, Technology Neutral Clean Electricity Credits, Carbon Sequestration Credit, Biofuels Credit, Advanced Manufacturing Credit; OBBBA relaxes calculations on land use and animal waste for the Biofuels Credit[22]	OBBBA adds metallurgical coal production credit to the Advanced Manufacturing Credit[22]	OBBBA does not mandate early phaseout of: Carbon Sequestration Credit, Advanced Manufacturing Credit (for solar and battery components), Advanced Energy Project Credit; OBBBA extends: Biofuels Credit[22]
Cross-cutting	OBBBA attempts to rescind USD 93.4 billion from IRA climate and environment programs[23]; DOE FY2026 budget	Executive Order on January 28, 2025 eliminates Community Benefits Plan requirements and		

	request for RD&D is 51% of the 2025 budget and is on par with funding levels of the mid-1980s and early 2000s[18]; Staff cuts orchestrated by the Department of Government Efficiency resulted in at least 25% of DOE staff resigning[23]	Justice40 social equity conditionalities as part of the administration's movement to end Diversity, Equity, and Inclusion activities[9]; DOE consolidates EERE, parts of OCED, parts of the Grid Deployment Office (GDO), the Office of State and Community Energy Programs (SCEP), the Office of Manufacturing and Energy Supply Chains (MESC), and the Federal Energy Management Program (FEMP) under CMEI[9]		

Supplementary References

1. IEA. ETP Clean Energy Technology Guide. (2025).
2. Joint Committee on Taxation. *Estimates Of Federal Tax Expenditures For Fiscal Years 2022-2026*. https://www.jct.gov/publications/2022/jcx-22-22/ (2022).
3. Joint Committee on Taxation. *Estimates Of Federal Tax Expenditures For Fiscal Years 2023-2027*. https://www.jct.gov/publications/2023/jcx-59-23/ (2023).
4. Joint Committee on Taxation. *Estimates of Federal Tax Expenditures for Fiscal Years 2024-2028*. https://www.jct.gov/publications/2024/jcx-48-24/ (2024).
5. Internal Revenue Service. SOI tax stats - Clean energy tax credit statistics. (2024).
6. Office of Tax Analysis. *Tax Expenditures - FY 2022*. https://home.treasury.gov/system/files/131/Tax-Expenditures-FY2022.pdf (2021).
7. Office of Tax Analysis. *Tax Expenditures - FY 2023*. https://home.treasury.gov/system/files/131/Tax-Expenditures-FY2023.pdf (2021).
8. Office of Tax Analysis. *Tax Expenditures - FY 2024*. https://home.treasury.gov/system/files/131/Tax-Expenditures-FY2024-update.pdf (2023).
9. Northey, H. & Marshall, C. Wright overhauls DOE, reflecting shift in US energy priorities. *E&E News by POLITICO* https://www.eenews.net/articles/wright-overhauls-doe-reflecting-shift-in-us-energy-priorities/ (2025).
10. Allsup, M. How DOE is using Biden-era funds to keep coal plants open. *Latitude Media* (2025).
11. Gallucci, M. How Trump gutted the team meant to build America’s energy future. *Canary Media* (2025).
12. Allsup, M. & Jenkins, L. M. The end of DOE as we know it. *Latitude Media* (2025).
13. Brigham, K. What’s Left of the LPO After the One Big Beautiful Bill? *Heatmap* (2025).

14. Urecki, Z. The Department of Energy's Recent Reorganization. *Bipartisan Policy Center* (2026).
15. The White House. Reinvigorating the Nuclear Industrial Base. (2025).
16. Pontecorvo, E. How the Loan Programs Office Became the Energy Dominance Financing Office. *Heatmap News* (2025).
17. Department of Energy. *FY 2026 Budget in Brief*. https://www.energy.gov/sites/default/files/2025-06/doe-fy-2026-bib-v6.pdf (2025).
18. Gallagher, K. S. & Anadón, L. D. DOE Budget Authority for Energy Research, Development, and Demonstration Database. (2025).
19. Executive Office of the President. Fiscal Year 2026 Discretionary Budget Request. (2025).
20. Department of Transportation, Federal Highway Administration. President Trump's Transportation Secretary Sean P. Duffy Unveils Revised NEVI Guidance to Allow States to Actually Build EV Chargers. (2025).
21. Pontecorvo, E. Trump Couldn't Kill Biden's EV Charging Program. *Heatmap* (2025).
22. Londagin, S., Zevin, A. & Bassett, L. A Brief Overview of Major US Clean Energy Tax Policy (2005-2025). (2025).
23. Allsup, M. 100 days of chaos at the Department of Energy. *Latitude Media* (2025).
