# Supplementary material for: Public institutions for cleantech commercialization in the United States
Source: Nat Commun. 2026 Aug 13;17:9725. doi: 10.1038/s41467-026-76484-6 (PMC13562745; doi:10.1038/s41467-026-76484-6)
Supplement: Supplementary file 2 [file 41467_2026_76484_MOESM2_ESM.pdf]

nature portfolio

Corresponding author(s): Jonas Meckling

Last updated by author(s): June 29, 2026

Reporting Summary

Nature Portfolio wishes to improve the reproducibility of the work that we publish. This form provides structure for consistency and transparency in reporting. For further information on Nature Portfolio policies, see our Editorial Policies and the Editorial Policy Checklist.

Statistics

For all statistical analyses, confirm that the following items are present in the figure legend, table legend, main text, or Methods section.

n/a	Confirmed	
☐	☒	The exact sample size (*n*) for each experimental group/condition, given as a discrete number and unit of measurement
☒	☐	A statement on whether measurements were taken from distinct samples or whether the same sample was measured repeatedly
☒	☐	The statistical test(s) used AND whether they are one- or two-sided<br>*Only common tests should be described solely by name; describe more complex techniques in the Methods section.*
☒	☐	A description of all covariates tested
☒	☐	A description of any assumptions or corrections, such as tests of normality and adjustment for multiple comparisons
☐	☒	A full description of the statistical parameters including central tendency (e.g. means) or other basic estimates (e.g. regression coefficient) AND variation (e.g. standard deviation) or associated estimates of uncertainty (e.g. confidence intervals)
☒	☐	For null hypothesis testing, the test statistic (e.g. *F*, *t*, *r*) with confidence intervals, effect sizes, degrees of freedom and *P* value noted<br>*Give P values as exact values whenever suitable.*
☒	☐	For Bayesian analysis, information on the choice of priors and Markov chain Monte Carlo settings
☒	☐	For hierarchical and complex designs, identification of the appropriate level for tests and full reporting of outcomes
☒	☐	Estimates of effect sizes (e.g. Cohen's *d*, Pearson's *r*), indicating how they were calculated

Our web collection on statistics for biologists contains articles on many of the points above.

Software and code

Policy information about availability of computer code

Data collection	Data was manually collected in Google Sheets.
Data analysis	Authors wrote custom code in R 4.5.1 to create the figures in the manuscript and supplementary information using dplyr, tidyr, and ggplot2. Authors conducted additional data exploration using waffle, fmsb, and ComplexUpset.

For manuscripts utilizing custom algorithms or software that are central to the research but not yet described in published literature, software must be made available to editors and reviewers. We strongly encourage code deposition in a community repository (e.g. GitHub). See the Nature Portfolio guidelines for submitting code & software for further information.

Data

Policy information about availability of data

All manuscripts must include a data availability statement. This statement should provide the following information, where applicable:

- Accession codes, unique identifiers, or web links for publicly available datasets
- A description of any restrictions on data availability
- For clinical datasets or third party data, please ensure that the statement adheres to our policy

Source data are provided with this paper. The raw institutional design dataset is currently under restricted access due to additional ongoing analysis by the authors.

Research involving human participants, their data, or biological material

Policy information about studies with human participants or human data. See also policy information about sex, gender (identity/presentation), and sexual orientation and race, ethnicity and racism.

Reporting on sex and gender	This research does not involve reporting on sex or gender.
Reporting on race, ethnicity, or other socially relevant groupings	This research does not involve reporting on race, ethnicity, or other socially relevant groupings.
Population characteristics	This research does not involve human participants, their data, or biological material. The subject of the study is institutions.
Recruitment	This research does not involve human participants, their data, or biological material. The subject of the study is institutions.
Ethics oversight	This research does not involve human participants, their data, or biological material. The subject of the study is institutions.

Note that full information on the approval of the study protocol must also be provided in the manuscript.

Field-specific reporting

Please select the one below that is the best fit for your research. If you are not sure, read the appropriate sections before making your selection.

☐ Life sciences ☒ Behavioural & social sciences ☐ Ecological, evolutionary & environmental sciences

For a reference copy of the document with all sections, see nature.com/documents/nr-reporting-summary-flat.pdf

Behavioural & social sciences study design

All studies must disclose on these points even when the disclosure is negative.

Study description	This study is a quantitative, cross-sectional, descriptive analysis of public institutions for cleantech commercialization in the United States active in 2024.
Research sample	The dataset includes the entire population of public institutions for cleantech commercialization in the United States active in 2024 that meet our inclusion criteria as described in the Methods. For inclusion in the dataset, institutions must meet six criteria. First, institutions must dedicate at least 20% of their activities or a minimum of USD 50 million annually to cleantech commercialization. Second, we include only programs or organizational units that shape or manage activities. An institution shapes or manages activities if it has dedicated staff or has published a strategic plan, annual report, or website that details its mission and approach. We also include institutions without a designated staff or strategy when they manage over USD 100 million in funding, as this indicates that there are likely dedicated management procedures, even if these are not openly published. Third, institutions must be public, meaning that they were established by the US government or by the US government in collaboration with private partners. Fourth, institutions must have been in existence as of 2024. Fifth, institutions must be relevant, which we distinguish by function. Institutions with a primary goal setting or coordination function are always coded, given that they meet the other inclusion criteria. Institutions with a primary external engagement function must have over 50 partners or network members. Institutions that primarily derisk through grants, other subsidies, and tax incentives must distribute over USD 10 million annually, while institutions that primarily derisk through loans, loan guarantees, or equity must distribute over USD 100 million annually. Finally, we only code institutions for which there is sufficient publicly available information to allow for confident coding of their functions. The dataset is representative of institutions meeting these criteria.
Sampling strategy	We collect data on the entire population rather than a sample. We identify institutions in the population by combing through legislation relevant to US green and cleantech industrial and commercialization policy, US government spending data, and the websites of government departments. In some cases, this led to snowball sampling, as one institution's materials may mention additional institutions. Sample size was not pre-determined. We included all institutions meeting the criteria, and stopped adding to the sample once we had examined the web infrastructure of every government department, every major piece of energy legislation from 2009-2024, and Department of Energy budget requests from 2020-2024.
Data collection	Coders reviewed an institution's website and public materials to evaluate each variable, with most information coming from workplans, annual reports, program and project descriptions, press releases, IRS guidelines {where applicable), and Notification of Funding Opportunity (NOFO) or Funding Opportunity Announcement (FOA) documents. Coders recorded the data using Google Sheets, leaving comments in each cell to justify their coding choices, and linking every cell to a PDF of the relevant source. All coding choices were reviewed by the supervisor.
Timing	Coders carried out data collection from January through December 2024.
Data exclusions	13 institutions were excluded from the dataset due to not meeting inclusion criteria on size/budget or sufficient publicly available information. Exclusion criteria were pre-established and are detailed in the Methods, as well as in the "Research sample" section above.
Non-participation	No participants dropped out, as this research did not involve human subjects.
Randomization	Participants were not assigned to treatment groups, as this was not experimental work and did not involve human subjects. Because

no experiments were performed, blinding researchers to experimental conditions or study hypotheses was not applicable.

Reporting for specific materials, systems and methods

We require information from authors about some types of materials, experimental systems and methods used in many studies. Here, indicate whether each material, system or method listed is relevant to your study. If you are not sure if a list item applies to your research, read the appropriate section before selecting a response.

Materials & experimental systems

n/a	Involved in the study
☒	☐ Antibodies
☒	☐ Eukaryotic cell lines
☒	☐ Palaeontology and archaeology
☒	☐ Animals and other organisms
☒	☐ Clinical data
☒	☐ Dual use research of concern
☒	☐ Plants

Methods

n/a	Involved in the study
☒	☐ ChIP-seq
☒	☐ Flow cytometry
☒	☐ MRI-based neuroimaging

Plants

Seed stocks

Report on the source of all seed stocks or other plant material used. If applicable, state the seed stock centre and catalogue number. If plant specimens were collected from the field, describe the collection location, date and sampling procedures.

Novel plant genotypes

Describe the methods by which all novel plant genotypes were produced. This includes those generated by transgenic approaches, gene editing, chemical/radiation-based mutagenesis and hybridization. For transgenic lines, describe the transformation method, the number of independent lines analyzed and the generation upon which experiments were performed. For gene-edited lines, describe the editor used, the endogenous sequence targeted for editing, the targeting guide RNA sequence (if applicable) and how the editor was applied.

Authentication

Describe any authentication procedures for each seed stock used or novel genotype generated. Describe any experiments used to assess the effect of a mutation and, where applicable, how potential secondary effects (e.g. second site T-DNA insertions, mosiacism, off-target gene editing) were examined.
