# Supplementary material for: Public institutions for cleantech commercialization in the United States
Source: Nat Commun. 2026 Aug 13;17:9725. doi: 10.1038/s41467-026-76484-6 (PMC13562745; doi:10.1038/s41467-026-76484-6)
Supplement: Supplementary file 3 [file 41467_2026_76484_MOESM3_ESM.pdf]

nature portfolio

Peer Review File

Public institutions for cleantech commercialization in the United States

Corresponding Author: Professor Jonas Meckling

This file contains all reviewer reports in order by version, followed by all author rebuttals in order by version.

Version 0:

Reviewer comments:

Reviewer #1

(Remarks to the Author)
This is a very interesting and well-written manuscript that has the potential to make an important contribution to current debates on the revival of industrial policy in the United States, particularly with respect to green industrial policy. The dataset on cleantech commercialization institutions constitutes a valuable resource that is likely to be of great interest to future research on the topic. I am therefore very supportive of the paper. That said, prior to publication, I encourage the authors to address several points that would improve the clarity and transparency of the data presentation and strengthen the overall contribution.

The creation of the dataset is a major strength of the paper. However, its content could be leveraged more systematically when introducing and discussing the categories used to classify institutions. In particular, the more frequent use of concrete examples illustrating the structure, functions, and roles of specific institutions would make the presentation easier to follow and more illustrative. For instance, it would be helpful to show how coordination operates within a specific institution, or how conditionality is applied in practice. This could be achieved either by selecting representative examples across different institutional types or by including a short vignette focused on a single institution.

The overall structure of the paper could be clarified. There are two relatively straightforward issues that, if addressed, would substantially improve accessibility. First, the manuscript would benefit from a brief roadmap paragraph explaining the logic of the section order. Second, the sequencing of sections is at times confusing. In particular, some material currently included in the section on the Trump administration and in the subsequent discussion might be more effectively reordered. It would be more intuitive to first discuss the functioning of institutions established prior to the second Trump administration, and then to examine how these arrangements may be altered or reoriented under the Trump administration.

The rationale for selecting the five functions performed by cleantech commercialization institutions should be introduced earlier in the manuscript. At present, it only becomes clear in the coding scheme section that these functions are derived from the existing literature, implying a largely deductive process. Clarifying this earlier—while still referring to the coding section—would help readers better understand the analytical framework from the outset.

Although my own expertise on U.S. industrial policy is secondary, I find that the paper sheds important light on a central but underexplored element of the Biden administration’s industrial policy agenda, namely institution building. This aspect is discussed relatively infrequently, particularly outside the United States, where attention tends to focus primarily on the scale of financial commitments under initiatives such as the Inflation Reduction Act. The manuscript’s contribution in this respect could be highlighted more explicitly.

Minor points:

While the choice of 2025 as the endpoint of the analysis is well justified, the rationale for selecting 1975 as the starting point could be discussed more explicitly.

The term “technology readiness level 4–10” (line 42) may not be accessible to a broad readership and should be briefly explained.

The example provided in lines 358–362 is particularly clear and informative. Including additional examples of this kind when discussing the different institutional functions would further enhance the exposition.

Overall, I appreciate the opportunity to read this interesting manuscript and wish the authors the best with the revisions.

Reviewer #2

(Remarks to the Author)
The way this paper traces the emergence of an “administrative state for green industrial policy” (p. 13) in the US is interesting, but the theoretical significance of these developments is severely underdeveloped. In its current form, it feels too descriptive for a high-end, peer-reviewed journal such as Nature Communications.

I found this opening hook at the bottom of page one particularly problematic:

“Yet relatively little is known about the design of public institutions aimed at accelerating technology commercialization beyond late industrializers in East Asia9. At the same time, research emphasizes that successful industrial policy hinges on the right institutions, though it is unclear which institutional designs work best in different contexts10.”

There are two issues here. First, political scientists and innovation scholars have written extensively about the design of public institutions and industrial policy for decades. Some of these (non-East Asian specialist) authors are cited in the bibliography (Fred Block, Fabio Bulfone, Erez Maggor, Linda Weiss). Others are not (Dan Breznitz, William Bonvillian, Rainer Kattel, Sean O’Riain). Regardless, it feels unfair to summarize the impact of their collective careers with “relatively little is known.”

Second and following from this point, the paper does little to engage with core concepts in their work. While the manuscript does engage with the literature on conditionality/discipline, there is no explicit discussion of the (1) the networked developmental state, (2) the primacy of the DOD in the US innovation system, and (3) the politicization of innovation.

Finally, in dismissing this well-developed literature, the paper stakes out too ambitious of a claim for itself, promising it “[improve] our systematic understanding of the institutions created and their effectiveness” at the top of p. 2. The paper does document the rise of an administrative (green industrial policy) state in the US, but I wouldn’t say that it delivers a “systematic” understanding of green industrial policy and it doesn’t say much of anything about effectiveness.

That is not to suggest that the paper has no theoretical merit. On the contrary, I see several possible theoretical contributions. But I think the front end of the paper needs show a bit more deference to the literature and identify a more narrowly targeted niche or set of gaps where it sees itself making a contribution.

I’ll offer one suggestion, although there may be more. I worry that the authors understate the importance of the DOD and the US military-industrial complex as a driver of green innovation policy and decarbonization. It could be that there methodology does not pick up on that because they are focused exclusively on organizations which are devoted to commercialization rather than adoption. Or perhaps the classified nature of DOD spending makes the military’s efforts in this space harder to observe.

I would acknowledge this limitation, but also use it as an opportunity to highlight one of the most surprising elements of this paper. An overwhelming number of scholars argue that the US’ industrial policy is driven by DOD and the broader military industrial complex (it’s not just Linda Weiss, one could add William Bonvillian, Zak Taylor, Stuart Leslie, Erica Fuchs, and even Fred Block when he’s writing about DARPA). It sounds like the US is changing in that we’re seeing the emergence of a large, visible industrial policy which stands outside the DOD. Side note: That post-2020 large, visible, non-defense related industrial policy may also explain why it has become more polarized.

I liked the discussion about how the US has done a better job of imposing conditions / disciplines than the existing literature recognizes. I thought that was an important step in the right direction, away from a purely descriptive exercise toward a more novel theoretical contribution.

The causal dynamics underpinning “coordination” and “external engagement” were a bit unclear to me. I didn’t object to the discussion (one can cover only so much in 23 pages), but the authors need to tread carefully here and might want to identify this as an avenue for future research. Specifically, to what extent does coordination reflect a conscious decision by the Biden administration, i.e. a reaction to evolving best practice in industrial policy? The focus on strategic planning sounds like it could have been imported from Europe’s 3S strategy. But it could also reflect the maturation of the technology. Or the practical need to weave together a growing alphabet soup of programs and organizations. Similarly, was external engagement driven by a networked developmental state-style logic, the evolving nature of the technology, a political calculus to secure buy-in from contested electoral districts, or partisan focus on organized labour or underprivileged communities? It’s hard to say. I think the paper would benefit from acknowledging and leaning into these limitations and using them to set up an agenda for future work.

Finally, I found the discussion of continuity and change under the Trump administration on p. 12 to be particularly fascinating. Instead of a full-scale partisan rollback, it sounds like elements of new this civilian industrial policy apparatus are here to stay. This strikes me another one of the paper’s stronger empirical claims, it reinforces the point above, and could represent a theoretical contribution in its own right in that it moves us beyond a discussion of industrial policy expansion

versus retrenchment to identify more nuanced patterns of institutional change (drawing, consciously or unconsciously, on the welfare state literature, e.g. Streeck and Thelen's 2005 book). I would keep or even expand this discussion.

To create space to do all of the above, I would encourage the authors to shorten or revise the discussion on pp. 13-14 which, at times, repeated claims from earlier in the manuscript. I would also move the methods section to the appendix, if possible. That would give the authors the page or so they need to (more fairly) identify narrower gaps or contested claims in the literature on industrial policy which they seek to contribute to, i.e. the idea that US industrial policy rests on the DOD and anything outside of it is (a) corporate welfare and (b) vulnerable to partisan rollback. The paper isn't quite there, but it could use the evidence it has collected to argue that, not only is the US catching up with industrial policy, but it's more disciplined and resilient than one would expect.

Oh, and one final point, is there any way to pair figure 1 with some figures on spending? I'm guessing it's been on an upward trajectory, but it would be nice to see some corroborating evidence on the substantive significance of this proliferation of programming.

Reviewer #3

(Remarks to the Author)
This is an interesting article that outlines five specific functions cleantech institutions perform and characterize them. I found that it contributes to our understanding of institutional design for the purpose of industrial policy purposes beyond conventional R&D institutions.

The article also provides a good understanding of the process of institutional building in the US and its recent evolution especially during the last and current administrations.

The methodology and analysis are sound. Although given that it is based on the stated goals of the institutions, it would have been helpful to know whether institutional formal design was consistent with the institutions' actual programs and operations (did they have enough means to achieve their goals? were programs/projects consistent with stated goals?) At the very least, the authors could have mentioned this as a limitation of their analysis.

I would have also liked the authors to outline their implications of their research for institutional design and outline a set of questions that emerge from their analysis. What does their research mean for US cleantech? other key areas of innovation? industrial-innovation policy more generally? They could also suggest future avenues for research. For instance, how does the US approach fare compare to that of China, the world's cleantech leader? Do their institutions pursue the same goals or others? How do their priorities differ? And how do those differences affect investment and project outcomes? Comparisons across different areas of innovation in the US would also be helpful, both in terms of understanding the extent to which the US has followed a consistent approach in different areas (or not) and if so why, and what that means.

Version 1:

Reviewer comments:

Reviewer #1

(Remarks to the Author)
Thank you for the opportunity to review the revised version of the manuscript. I am satisfied with the way the authors addressed my comments and believe that the manuscript has the potential to make an important contribution to the debate on new forms of industrial policy in the United States, while also potentially stimulating the development of comparative databases covering other high-income economies.

The article has improved considerably, particularly with regard to the presentation and accessibility of the dataset, which was my main concern in the previous version. I am therefore happy to recommend publication.

Reviewer #2

(Remarks to the Author)
I like the way the authors have reframed this study to trace the emergence of a "visible" developmental state associated with the DOE. That satisfies many of my concerns and strikes me as an original and valuable theoretical contribution to a literature which has, to date, focused on a "hidden" industrial policy run through DOD or "small" programs such as SBIR (Keller and Block 2013). While I would like to see the authors engage even more fully with the literature to highlight the novelty of this finding and dig into the causal factors behind this, I understand that this is not appropriate for Nature Communications and should be left for future research. That's fine, although I found the research agenda on lines 582-596 rather high level and a bit unsatisfying. For example, the authors don't directly engage any of my questions and they don't mention China by name (reviewer 3). I don't think the authors should be bound by our comments, but could they sharpen the agenda and be more specific about the two or three most important questions, puzzles, causal claims, or cases that we need to investigate in light of this research? Overall, I'm happy to accept this for publication in Nature Communications, but I think the research agenda on lines 582-596 would benefit from a second look.

Reviewer #3

(Remarks to the Author)
I am satisfied with the way the authors reviewed my comments and I am happy to recommend the paper's publication.

Open Access This Peer Review File is licensed under a Creative Commons Attribution 4.0 International License, which permits use, sharing, adaptation, distribution and reproduction in any medium or format, as long as you give appropriate credit to the original author(s) and the source, provide a link to the Creative Commons license, and indicate if changes were made.
In cases where reviewers are anonymous, credit should be given to 'Anonymous Referee' and the source.
The images or other third party material in this Peer Review File are included in the article's Creative Commons license, unless indicated otherwise in a credit line to the material. If material is not included in the article's Creative Commons license and your intended use is not permitted by statutory regulation or exceeds the permitted use, you will need to obtain permission directly from the copyright holder.
To view a copy of this license, visit https://creativecommons.org/licenses/by/4.0/

Dear Reviewers,

Thank you for your valuable comments. We felt they were constructive and helped strengthen and sharpen the manuscript significantly. We here provide point-by-point responses to all comments.

Thank you again for the constructive engagement with our manuscript.

The authors

Reviewer Comments

Reviewer #1:

1.1 Comment

This is a very interesting and well-written manuscript that has the potential to make an important contribution to current debates on the revival of industrial policy in the United States, particularly with respect to green industrial policy. The dataset on cleantech commercialization institutions constitutes a valuable resource that is likely to be of great interest to future research on the topic. I am therefore very supportive of the paper. That said, prior to publication, I encourage the authors to address several points that would improve the clarity and transparency of the data presentation and strengthen the overall contribution.

Response

Thank you for your encouraging comments and for recognizing the potential contribution of the dataset to future research. We appreciate your suggestions for improving the clarity and transparency of the paper and address each comment individually below.

1.2 Comment

The creation of the dataset is a major strength of the paper. However, its content could be leveraged more systematically when introducing and discussing the categories used to classify institutions. In particular, the more frequent use of concrete examples illustrating the structure, functions, and roles of specific institutions would make the presentation easier to follow and more illustrative. For instance, it would be helpful to show how coordination operates within a specific institution, or how conditionality is applied in practice. This could be achieved either by selecting representative examples across different institutional types or by including a short vignette focused on a single institution.

Response

Thank you for your suggestion. We have added additional examples to illustrate goal setting (lines 245-247) and coordination (lines 250-253). We have also included more details about how conditionalities may be applied (lines 333-335) and the forms external engagement can take (lines 433-435).

<u>1.3 Comment</u>
The overall structure of the paper could be clarified. There are two relatively straightforward issues that, if addressed, would substantially improve accessibility. First, the manuscript would benefit from a brief roadmap paragraph explaining the logic of the section order. Second, the sequencing of sections is at times confusing. In particular, some material currently included in the section on the Trump administration and in the subsequent discussion might be more effectively reordered. It would be more intuitive to first discuss the functioning of institutions established prior to the second Trump administration, and then to examine how these arrangements may be altered or reoriented under the Trump administration.

Response
Thank you for pointing this out. We have added a brief roadmap of the paper (lines 78–82). Regarding the sequencing of the Trump section and the discussion, we believe the paper should conclude with the discussion in order to summarize the full set of findings, including institutional change under Trump. To clarify the chronology, we added a sentence at the beginning of the discussion (lines 528–530).

<u>1.4 Comment</u>
The rationale for selecting the five functions performed by cleantech commercialization institutions should be introduced earlier in the manuscript. At present, it only becomes clear in the coding scheme section that these functions are derived from the existing literature, implying a largely deductive process. Clarifying this earlier—while still referring to the coding section—would help readers better understand the analytical framework from the outset.

Response
Thank you for noting this. We have added a clarification of how the functions were derived (lines 71–73).

<u>1.5 Comment</u>
Although my own expertise on U.S. industrial policy is secondary, I find that the paper sheds important light on a central but underexplored element of the Biden administration's industrial policy agenda, namely institution building. This aspect is discussed relatively infrequently, particularly outside the United States, where attention tends to focus primarily on the scale of financial commitments under initiatives such as the Inflation Reduction Act. The manuscript's contribution in this respect could be highlighted more explicitly.

Response
Thank you for suggesting to better highlight institution-building. Reviewer 2 suggested that we specifically point out the rise of a "visible developmental state" centered around the DOE, while previous work emphasized a more hidden industrial policy apparatus in the DOD. We now emphasize this finding in the "Growth, governance, and scope" section (lines 165-170) and in the Discussion (lines 534-537).

Minor points:

1.6 Comment
While the choice of 2025 as the endpoint of the analysis is well justified, the rationale for selecting 1975 as the starting point could be discussed more explicitly.

Response
Thank you for recommending this clarification. Our dataset captures public institutions supporting cleantech commercialization that were active in 2024. The year 1975 refers to the founding year of the oldest institution in the dataset. We added a sentence clarifying this in lines 61-64.

1.7 Comment
The term "technology readiness level 4–10" (line 42) may not be accessible to a broad readership and should be briefly explained.

Response
Thank you for raising this point. We have added a short explanation explaining how technology readiness levels 4-10 map onto the innovation process in lines 58-60.

1.8 Comment
The example provided in lines 358–362 is particularly clear and informative. Including additional examples of this kind when discussing the different institutional functions would further enhance the exposition.

Response
Thank you for this feedback. We have added some additional examples as addressed in Comment 1.2.

1.9 Comment
Overall, I appreciate the opportunity to read this interesting manuscript and wish the authors the best with the revisions.

Response
Many thanks for your positive comments and for the helpful suggestions to improve the paper.

Reviewer #2:

2.1 Comment
The way this paper traces the emergence of an "administrative state for green industrial policy" (p. 13) in the US is interesting, but the theoretical significance of these developments is severely underdeveloped. In its current form, it feels too descriptive for a high-end, peer-reviewed journal such as Nature Communications.

I found this opening hook at the bottom of page one particularly problematic:

“Yet relatively little is known about the design of public institutions aimed at accelerating technology commercialization beyond late industrializers in East Asia9. At the same time, research emphasizes that successful industrial policy hinges on the right institutions, though it is unclear which institutional designs work best in different contexts10.”

There are two issues here. First, political scientists and innovation scholars have written extensively about the design of public institutions and industrial policy for decades. Some of these (non-East Asian specialist) authors are cited in the bibliography (Fred Block, Fabio Bulfone, Erez Maggor, Linda Weiss). Others are not (Dan Breznitz, William Bonvillian, Rainer Kattel, Sean O’Riain). Regardless, it feels unfair to summarize the impact of their collective careers with “relatively little is known.”

Response
This comment has been helpful to more clearly position our contribution in the broader literature.

We were familiar with the work of most of the authors listed but carefully reviewed their work again. We have revised our contribution statement to say that little is known about the design of institutions for green industrial policy specifically (lines 31-32; 50-51). We cite the political economy and economic sociology literature on industrial policy as showing that institutional design matters for the effectiveness of industrial policy (lines 32-34). We agree that this body of work has made important contributions to our understanding of institutions and industrial policy. At the same time, the existing literature is based on case studies. By contrast, our systematic, bottom-up mapping of institutions offers a larger-N understanding of patterns of institutional design. So, our contribution is to examine a new area of industrial policy, i.e., green industrial policy, and to adopt a new approach to data collection.

We appreciate your suggestion to further develop our theoretical contribution. We have centered that contribution around the rise of a visible developmental state, with the DOE as its anchor. At the same time, the style of articles at Nature Communications does not allow for an extended theoretical development as in the case of political science articles. We, therefore, infuse our argument in the empirical section and the Discussion section (lines 165-170; 534-537).

2.2 Comment
Second and following from this point, the paper does little to engage with core concepts in their work. While the manuscript does engage with the literature on conditionality/discipline, there is no explicit discussion of the (1) the networked developmental state, (2) the primacy of the DOD in the US innovation system, and (3) the politicization of innovation.

Response
Thank you for raising this point. We have added an acknowledgement of the DOD’s primacy in the US innovation system and its connection to the politicization of innovation, noting that the opacity of many DOD activities allowed commercialization support to persist despite political

opposition to industrial policy (lines 165–170). We also added a clearer reference to the developmental network state (lines 426-429), a concept that heavily informed the“network building” variable in our dataset.

<u>2.3 Comment</u>
Finally, in dismissing this well-developed literature, the paper stakes out too ambitious of a claim for itself, promising it “[improve] our systematic understanding of the institutions created and their effectiveness” at the top of p. 2. The paper does document the rise of an administrative (green industrial policy) state in the US, but I wouldn’t say that it delivers a “systematic” understanding of green industrial policy and it doesn’t say much of anything about effectiveness.

That is not to suggest that the paper has no theoretical merit. On the contrary, I see several possible theoretical contributions. But I think the front end of the paper needs show a bit more deference to the literature and identify a more narrowly targeted niche or set of gaps where it sees itself making a contribution.

Response
Thank you, this relates to comment 2.1. We now clearly cite the small but important body of work on institutions and industrial policy as having shown that institutional design matters for industrial policy effectiveness (lines 32-34).

We position our contribution as systematically mapping US institutions for green industrial policy specifically and demonstrating the rise of a “visible green developmental state.” We maintain the word “systematic” as we apply a rigorous coding scheme with 129 dimensions to 122 institutions. This type of data collection has not yet been conducted on institutions for industrial policy in general, as far as we know. Earlier work in political economy and economic sociology focused on case studies that examined a small set or individual institutions.

We agree that our paper does not speak to effectiveness. The cited sentence made a general statement on the knowledge gaps. Our paper is descriptive and conducts the mapping of institutional design. The new language in the introduction clearly conveys that (lines 50-51).

<u>2.4 Comment</u>
I’ll offer one suggestion, although there may be more. I worry that the authors understate the importance of the DOD and the US military-industrial complex as a driver of green innovation policy and decarbonization. It could be that there methodology does not pick up on that because they are focused exclusively on organizations which are devoted to commercialization rather than adoption. Or perhaps the classified nature of DOD spending makes the military’s efforts in this space harder to observe.

I would acknowledge this limitation, but also use it as an opportunity to highlight one of the most surprising elements of this paper. An overwhelming number of scholars argue that the US’ industrial policy is driven by DOD and the broader military industrial complex (it’s not just Linda Weiss, one could add William Bonvillian, Zak Taylor, Stuart Leslie, Erica Fuchs, and even Fred Block when he’s writing about DARPA). It sounds like the US is changing in that

we're seeing the emergence of a large, visible industrial policy which stands outside the DOD. Side note: That post-2020 large, visible, non-defense related industrial policy may also explain why it has become more polarized.

Response
Thank you for these two points.

First, we now explicitly acknowledge the limitation regarding the representation of DOD institutions in our dataset in the Methods (lines 776-781). Because much DOD spending is classified, our dataset—based on publicly available materials—likely omits some cleantech-relevant activity within the DOD.

Second, we appreciate the nudge to more clearly highlight a key finding: the rise of a "visible developmental state" centered around the DOE. We now highlight this in the section on "Growth, governance, and scope" (lines 165-170) and in the Discussion (lines 534-537).

2.5 Comment
I liked the discussion about how the US has done a better job of imposing conditions / disciplines than the existing literature recognizes. I thought that was an important step in the right direction, away from a purely descriptive exercise toward a more novel theoretical contribution.

Response
Thank you for this comment and for recognizing our finding that discipline/conditionalities in the US are more prevalent than the literature typically suggests. We appreciate your observation that this helps move the paper beyond a purely descriptive account toward a stronger theoretical contribution.

2.6 Comment
The causal dynamics underpinning "coordination" and "external engagement" were a bit unclear to me. I didn't object to the discussion (one can cover only so much in 23 pages), but the authors need to tread carefully here and might want to identify this as an avenue for future research. Specifically, to what extent does coordination reflect a conscious decision by the Biden administration, i.e. a reaction to evolving best practice in industrial policy? The focus on strategic planning sounds like it could have been imported from Europe's 3S strategy. But it could also reflect the maturation of the technology. Or the practical need to weave together a growing alphabet soup of programs and organizations. Similarly, was external engagement driven by a networked developmental state-style logic, the evolving nature of the technology, a political calculus to secure buy-in from contested electoral districts, or partisan focus on organized labour or underprivileged communities? It's hard to say. I think the paper would benefit from acknowledging and leaning into these limitations and using them to set up an agenda for future work.

Response
Our paper does not make causal claims. At times, we offer potential explanations of specific descriptive findings but are careful to frame these as hypotheses. The reviewer raises interesting potential explanations for specific trends in institutional design. We do not have the space to engage in hypothesizing all descriptive findings. But we followed the suggestion to add this as an area for future research. We added a full paragraph on research implications to the Discussion (lines 582-596), before turning to policy implications.

2.7 Comment
Finally, I found the discussion of continuity and change under the Trump administration on p. 12 to be particularly fascinating. Instead of a full-scale partisan rollback, it sounds like elements of new this civilian industrial policy apparatus are here to stay. This strikes me another one of the paper's stronger empirical claims, it reinforces the point above, and could represent a theoretical contribution in its own right in that it moves us beyond a discussion of industrial policy expansion versus retrenchment to identify more nuanced patterns of institutional change (drawing, consciously or unconsciously, on the welfare state literature, e.g. Streeck and Thelen's 2005 book). I would keep or even expand this discussion.

Response
We appreciate that this analysis is interesting. We had considered using Kathleen Thelen's typology of institutional change, including displacement, layering, drift and conversion, but decided against it. It didn't map well onto what we observe and would have introduced more concepts that needed explaining for a broad, interdisciplinary audience. That said, we agree this points to an important line of inquiry on institutional change, but do not pursue it here to maintain focus.

2.8 Comment
To create space to do all of the above, I would encourage the authors to shorten or revise the discussion on pp. 13-14 which, at times, repeated claims from earlier in the manuscript. I would also move the methods section to the appendix, if possible. That would give the authors the page or so they need to (more fairly) identify narrower gaps or contested claims in the literature on industrial policy which they seek to contribute to, i.e. the idea that US industrial policy rests on the DOD and anything outside of it is (a) corporate welfare and (b) vulnerable to partisan rollback. The paper isn't quite there, but it could use the evidence it has collected to argue that, not only is the US catching up with industrial policy, but it's more disciplined and resilient than one would expect.

Response
Thank you for this recommendation. We have some flexibility within the word limit and therefore do not need to make the suggested cuts. In addition, the Methods section does not count toward the word limit, so there is no need to move it to the appendix. This allows us to incorporate some of the additions you suggested. In particular, we now note the historical primacy of the DOD and the hidden developmental state, and signal that the paper documents the rise of a more visible industrial policy state (lines 165-170; 534-537).

<u>2.9 Comment</u>
Oh, and one final point, is there any way to pair figure 1 with some figures on spending? I'm guessing it's been on an upward trajectory, but it would be nice to see some corroborating evidence on the substantive significance of this proliferation of programming.

Response
Thank you for this suggestion. Unfortunately, we are not able to construct a comparable figure on spending. Budget data are typically not reported at a level of granularity that would allow us to isolate funding for the commercialization institutions captured in our dataset from R&D activities. In addition, while our dataset identifies all relevant institutions active in 2024, constructing a time series of spending would require identifying the full universe of relevant institutions in each year, including those that no longer exist. This would require substantial additional data collection and is beyond the scope of the paper.

Reviewer #3:

<u>3.1 Comment</u>
This is an interesting article that outlines five specific functions cleantech institutions perform and characterize them. I found that it contributes to our understanding of institutional design for the purpose of industrial policy purposes beyond conventional R&D institutions.

The article also provides a good understanding of the process of institutional building in the US and its recent evolution especially during the last and current administrations.

Response
Thank you for your positive assessment of the manuscript and for recognizing its contribution to understanding institutional design and institution-building.

<u>3.2 Comment</u>
The methodology and analysis are sound. Although given that it is based on the stated goals of the institutions, it would have been helpful to know whether institutional formal design was consistent with the institutions' actual programs and operations (did they have enough means to achieve their goals? were programs/projects consistent with stated goals?) At the very least, the authors could have mentioned this as a limitation of their analysis.

Response
Thank you for pointing this out. Because our dataset relies on publicly available materials, we do not have access to third-party evaluations for most institutions. Our coding therefore reflects institutions' stated goals and activities rather than verified implementation. We have clarified this limitation in the Methods, lines 773–776.

3.3 Comment

I would have also liked the authors to outline their implications of their research for institutional design and outline a set of questions that emerge from their analysis. What does their research mean for US cleantech? other key areas of innovation? industrial-innovation policy more generally? They could also suggest future avenues for research. For instance, how does the US approach fare compare to that of China, the world's cleantech leader? Do their institutions pursue the same goals or others? How do their priorities differ? And how do those differences affect investment and project outcomes? Comparisons across different areas of innovation in the US would also be helpful, both in terms of understanding the extent to which the US has followed a consistent approach in different areas (or not) and if so why, and what that means.

Response

We appreciate the suggestion to develop a research agenda. We now do so in a new paragraph in the Discussion section. We hone in on comparative lessons across economies, the origins of institutional design, and the effectiveness of institutions (lines 582-596).
